# Donald O. Hebb and the Organization of Behavior: 17 years in the writing

**DOI:** 10.1186/s13041-020-00567-8

**Published:** 2020-04-06

**Authors:** Richard E. Brown

**Affiliations:** Department of Psychology and Neuroscience, Dalhousie University, Halifax, Nova Scotia B3H 4R2 Canada

## Abstract

*The Organization of Behavior* has played a significant part in the development of behavioural neuroscience for the last 70 years. This book introduced the concepts of the “Hebb synapse”, the “Hebbian cell assembly” and the “Phase sequence”. The most frequently cited of these is the Hebb synapse, but the cell assembly may be Hebb’s most important contribution. Even after 70 years, Hebb’s theory is still relevant because it is a general framework for relating behavior to synaptic organization through the development of neural networks. *The Organization of Behavior* was Hebb’s 40th publication. His first published papers in 1937 were on the innate organization of the visual system and he first used the phrase “the organization of behavior” in 1938. However, Hebb wrote a number of unpublished papers between 1932 and 1945 in which he developed the ideas published in *The Organization of Behavior*. Thus, the concept of the neural organization of behavior was central to Hebb’s thinking from the beginning of his academic career. But his thinking about the organization of behavior in 1949 was different from what it was between 1932 and 1937. This paper examines Hebb’s early ideas on the neural basis of behavior and attempts to trace the rather arduous series of steps through which he developed these ideas into the book that was published as *The Organization of Behavior*. Using the 1946 typescript and Hebb’s correspondence we can see a number of changes made in the book before it was published. Finally, a number of issues arising from the book, and the importance of the book today are discussed.

## Introduction

This meeting celebrates the 70th anniversary of the publication of *The Organization of Behavior* by Donald O. Hebb [77]. Since its publication, *The Organization of Behavior* has become one of the most influential books in Psychology and Neuroscience (over 31,200 Google scholar citations in January 2020). In 2017 Dick Passingham named *The Organization of Behavior* one of the top 5 books in Cognitive neuroscience^1^ because Hebb’s “ideas have turned out to be incredibly powerful in understanding how the brain actually works” [44]. According to Adams ([2], page 419), Hebb’s *Organization of Behavior* and Darwin’s *On the Origin of Species* are two of the most influential books in the history of biology. Because Hebb’s book had been out of print since 1966, Peter Milner and I had it reprinted by Lawrence Erlbaum in 2002. In our Foreword to this edition [22], we included a list of Hebb’s publications and a list of biographies and obituaries of Hebb. This paper will focus on the work done by Hebb between 1932 and 1949, which led to the publication of *The Organization of Behavior*.

*The Organization of Behavior* introduced the concepts of the “Hebb synapse”, the “Hebbian cell assembly” and the “Phase Sequence”. While the Hebb synapse has become the most cited, and “better known than Donald Hebb himself” [166], the cell assembly may be his most lasting legacy [41, 97, 122, 153, 163]. Research on the phase sequence has lagged behind, but multi-electrode recording techniques have enabled researchers to investigate the integration of cell assemblies into larger phase sequences [4, 129]. *The Organization of Behavior* remains influential because it continues to stimulate research in many areas of neuroscience including studies of learning and memory; the long-term effects of environment on development; aging; computer modeling of the brain, robotics, and artificial intelligence [21]. This meeting is evidence of Hebb’s continued influence on research into synaptic function in learning and memory. This paper describes the long and arduous process that Hebb went through in order to write and publish *The Organization of Behavior.* It uses unpublished notes, papers, letters and a typescript of the first draft to show the development of the ideas that went into this seminal book.

## A brief overview of Hebb’s life and early education (Table 1)

Hebb [81, 83] published two autobiographical papers and details of Hebb’s life and work are given in previous papers [18, 19, 23], so only a brief overview is given here. Hebb’s father, Arthur Morrison Hebb, and his mother, Mary Clara Olding, both received their medical degrees from Dalhousie University and were physicians in the village of Chester, Nova Scotia. Donald was born on 22 July 1904, the first of four children. His brother Andrew (1905–2005) received a law degree from Dalhousie and went into business. Peter (1909–1955) was a physician and Catherine (1912–1978) received her PhD in Physiology from McGill University, studying the physiology of the digestive system with Dr. Boris Babkin. Later she won a scholarship to Edinburgh University and did research on the biosynthesis of acetylcholine at the Institute of Animal Physiology at Babraham, Cambridge [56].

Hebb went to the Chester School until grade 11 and then completed high school at the Halifax County Academy before entering Dalhousie University in Halifax in 1921. He majored in English and Philosophy, with the intention of becoming a novelist [83]. During the time that he was an undergraduate, Psychology was taught in the Philosophy Department and in the 1922–23 academic year Hebb took Philosophy 1, “Logic and Psychology” from Professor H.L. Stewart (who wrote *Questions of the day in philosophy and psychology,* in 1912). According to the *Calendar of Dalhousie University, 1921–22*, the textbooks for this class were *An Introductory Logic* by James Edwin Creighton (1919), and the *Textbook of Psychology* by William James (1892), with references made to *The Principles of Psychology* by William James (1890) and *An Introduction to Social Psychology* by William McDougall (1908).

In 1924–25, Hebb took Philosophy 8 “Philosophic ideas in Literature “ with Professor H.L. Stewart, a course which included “a study of philosophic ideas in Tolstoy, Hardy, Anatole France, H.G. Wells, Ibsen, Morley, Fredric Harrison, Mrs Humphrey Ward, Rabindranath Tragore, Wilfred Ward, George Meredith” (*Calendar of Dalhousie University, 1924–25*). In the same year he took Philosophy 9 “Experimental Psychology”, from Professor N.J. Symons, in which the textbook was *An Elementary Laboratory Course in Psychology* by H.S. Langfeld and F. H. Allport (1916). Thus, Hebb had taken 3 courses that contained topics in Psychology while completing his B.A., even though he took no Psychology courses per se.

## Hebb’s M.A. Thesis [58]

After graduating from Dalhousie in 1925, Hebb obtained a teaching certificate from the Provincial Normal College in Truro, Nova Scotia, and became the principal of his old school in Chester for a year. He then moved to Montreal, where he was a teacher and a part-time graduate student in Psychology at McGill University under the supervision of Professor Chester Kellogg. During this time, he started an educational experiment in his school as he found that students of all intellectual abilities were failing. He decided to change the school procedures to facilitate education, giving students no homework and no punishment for inattention. He persuaded the children that school-work was a privilege, gave them interesting things to do in class and sent any who disrupted the class outside to play [57].

As a graduate student at McGill University, he took a course in experimental psychology and seminars in systematic psychology, with a minor in Education. For his M.A. thesis, he studied Sherrington’s *Integrative activity of the Nervous System* [170] and Pavlov’s *Conditioned Reflexes* [149], and wrote a theoretical M.A. thesis entitled *Conditioned and Unconditioned Reflexes and Inhibition* [58]. In 1980, Hebb wrote that “My M.A. thesis, written in bed [while he was ill with a tubercular hip] tried to show that skeletal reflexes are a product of intra-uterine learning. This was nonsense, but no immediate disproof was available at the time” ([83], page 282–283). However, a reading of Hebb’s M.A. thesis shows that this comment is untrue. As stated in the Introduction to this thesis:

“The purpose of this paper is to present a theory of the functioning of the synapse based on the experimental work of Sherrington and Pavlov, on reflexes and inhibitions.

The implications of these things for psychological theory, in some aspects, has been far from clear. In looking for a firm basis for psychology in physiology, there are some peculiarities about the results of both investigators which demand serious consideration and suggest another interpretation of their work.” ([58], page 2).

**Fig. 1:**
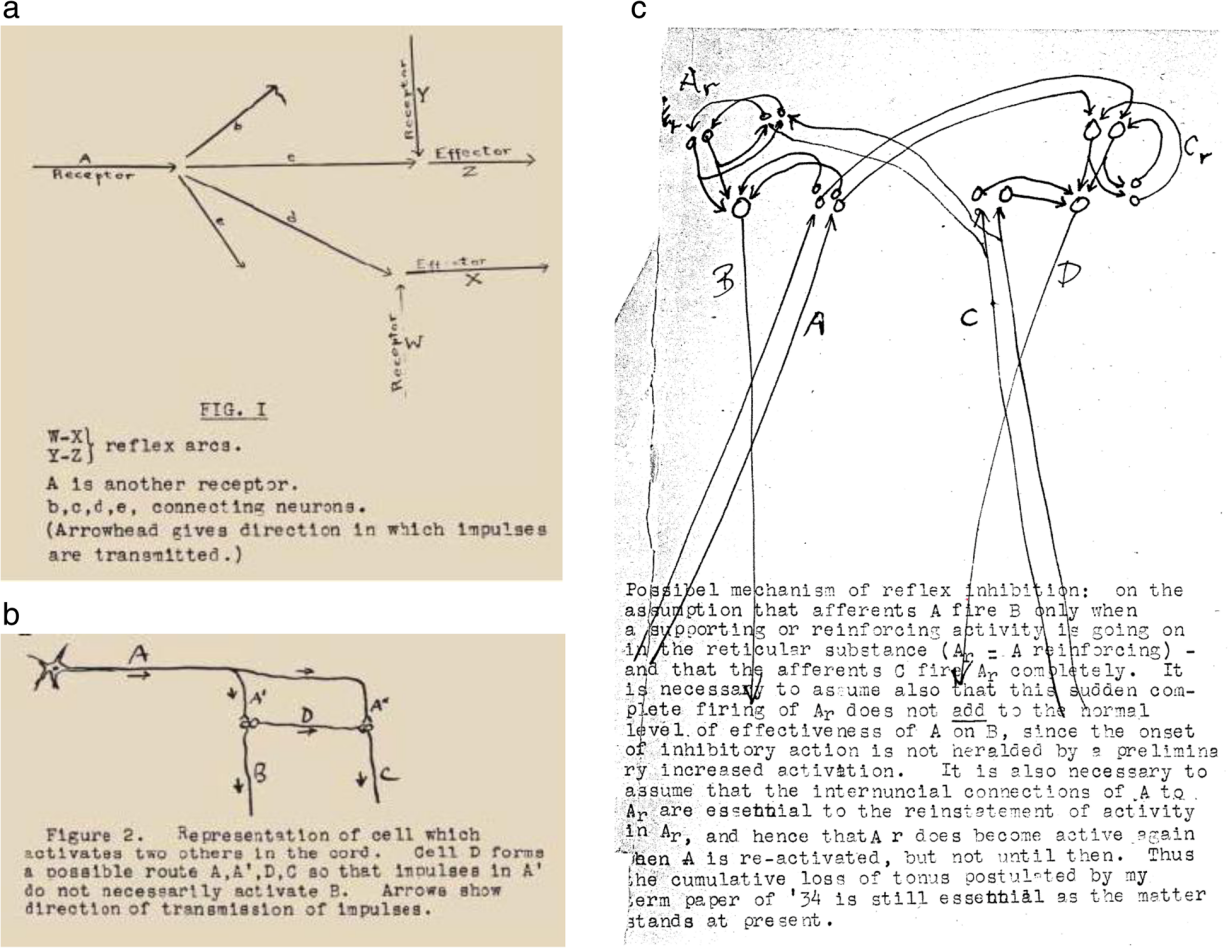
Hebb’s early concepts of synaptic change during learning. (**a**) Figure 1 from Hebb's MA thesis [58] shows his early concept of the synaptic changes underlying conditioning. (**b**) Figure 2 from [59] shows two possible routes of synaptic activity. An axon A with two terminal branches A’ and A” activates B and C. With greater activity of B, the route A’–B will be strengthened, but if there is an interneuron D, which is activated by A’, the route A’–D–C will be strengthened. (**c**) A possible mechanism of reflex inhibition. This figure was attached to Hebb’s 1934 essay [59]. It shows that his concept of inhibitory synapses was well developed in 1934, but was later deleted from his theory. **a**, **b**, **c**: Reprinted from unpublished papers of D.O Hebb held in McGill University Archives, Montreal, Quebec, file MG1045 [58, 59]. Permission has been obtained from Mary Ellen Hebb to reprint them. **a** and **b** are also in: Brown RE, Milner PM. The legacy of Donald O. Hebb: more than the Hebb synapse. Nat Rev Neurosci. 2003;4:1013–9 [23]. Ownership of copyright in original research articles remains with the author, Richard E. Brown

There is nothing about intra-uterine learning in this thesis. The four chapter headings are (I) Functioning of the synapse in the conditioned reflex, (II) The unconditioned reflex, (III) Unconditioned inhibition and (IV) Conditioned inhibition: hypnosis, sleep, and the waking state. Chapter 1 contains Hebb’s first illustration of his idea of the synaptic changes associated with conditioning (see Fig. 1a). This figure shows branches of a stimulus input going to two unconditioned reflex arcs. Pavlov showed that if one of the reflex arcs is active when a stimulus is applied to the input, the branch going to that reflex becomes more potent; branches going to inactive effectors do not. Hebb summarized this by stating that: “An excited neuron tends to decrease its discharge to inactive neurons, and increase this discharge to any active neuron, and therefore to form a route to it, whether there are intervening neurons between the two or not. With repetition this tendency is prepotent in the formation of neural routes.” ([58], page 13). This was the first description of Hebb’s theory of synaptic function in learning.

**Table 1:**
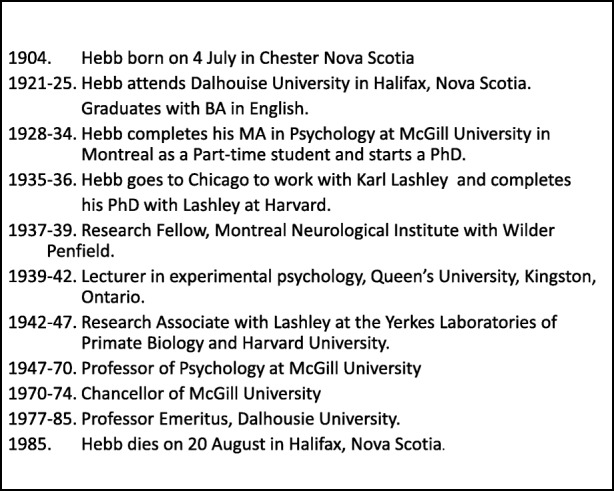
A Brief Time-Line of Hebb’s Career

At the end of section II of this thesis, Hebb states that: “There are only two types of simple unconditioned reflex, though these may combine in more complex forms: (1) the reaction such as to increase stimulation (positive) and (2) such as to decrease it. This is a feature of reflex activity which has not been taken account of before as a general characteristic of all reflexes, and it suggests a genesis of the reflex as due to environment just as the laboratory conditioned reflex is due to an experimenter.” ([58], page 28).

One of the important features of this thesis was the discussion of unconditioned and conditioned inhibition. On unconditioned inhibition, he says: “In reciprocal inhibition it is noteworthy that it occurs with reflexes which could not function simultaneously even if one of them were not inhibited. Flexion inhibits extension, but if this were not so the two reactions still could not take place at the same time. This suggests strongly that inhibition is not established by heredity but by the actual functioning of the organism itself; a result of the opposition of two reflexes.” (page 31). The summary statement of the thesis (page 60–62) is as follows:

“Supposing the individual synapse to obey the same general laws in all neural activity, an analysis of Pavlov’s work on the conditioned reflex gives the following generalization: An excited neuron tends to decrease its discharge to inactive neurons and increase this discharge to any active neuron, tending to form a route to it. With repetition this tendency is prepotent in the formation of neural routes.”

“The unconditioned reflex appears then as a reflex conditioned by the environment. This occurs in two ways, according to the kind of reaction. The unconditioned reflex may be grouped into (1) those reflexes whose reaction increases the stimulus, as the exterior thrust, or muscle tone, (2) those whose reaction decreases the stimulus, which is strong or persistent, as the salivary reflex to HCl or sand placed in the mouth, and (3) those which may combine (1) and (2) in varying ways, as the scratch reflex. If the generalization of neural action above is sound, these reflexes may be regarded as the result of the functioning of the law which established the conditioned reflex in the laboratory, under the interaction of environment and organism only. “Similarly the inhibition of the spinal cord may, under this generalization, be a conditioning by environment, in the interaction of the spinal reflexes. Activity of the opposed effectors would facilitate a re-routing of the impulses from the inhibited receptor so that the inhibited effector is not excited. The exact adjustment of this process is effected by the repeated opposition of the two reflexes, until synaptic block between inhibited receptor and inhibiting effector is lowered to the point that impulses may follow this route whenever the inhibiting effector is more active than the inhibited effector. The latter thereupon loses tone because of the change of route of impulses from the corresponding (inhibited) receptors.”

“This account of unconditioned inhibition seems to be considerably more satisfactory than the rather improbable refractory-phase hypothesis. [The hypothesis that inhibition is due to the development of a prolonged refractory phase in the tissues concerned. See Telford [178]]. Together with a simplified account of the unconditioned reflex, this is argument for giving serious consideration to the generalization above. Applying it to conditioned inhibition adds further support.”

“Here also inhibition appears as a re-routing of the nervous activity. The inhibiting agent is the constantly active reflexes of posture, breathing, and of visceral activity generally. This means that upon establishment of an internal inhibition these activities (postural and visceral) would be re-inforced, but this in a localized inhibition would be too weak to be discernable. When inhibition becomes general, however, as in hypnosis, this re-inforcement becomes very evident, in the catalepsy of the animal: a very strong maintenance of the posture. ([58], page 60–62).”

These detailed quotations show that this thesis had nothing to do with intra-uterine learning and that Hebb had seriously considered the importance of inhibition as well as excitation in the reflex arc. Thus, the ideas for the Hebb synapse began with Hebb’s M.A. thesis and these ideas developed and changed over the next 17 years.

## Hebb’s unpublished paper on neural action [59]

After the completion of his M.A. thesis, Hebb started his PhD research at McGill on classical conditioning with Drs. Boris Babkin and Leonid Andreyev, both of whom had worked with Pavlov in St. Petersburg. Hebb, however, became disillusioned with Pavlovian conditioning procedures and his graduate studies, and left McGill in the fall of 1934 to complete his PhD with Karl Lashley at the University of Chicago [83]. Lashley had just published his book *Brain Mechanisms of Intelligence* [112] and his American Psychological Association presidential address outlined the state of the art of physiological psychology [113]. After he moved to Chicago, Hebb became part of a group of exceptional psychobiology students that studied with Lashley. These included Frank Beach, David Krech, Norman Maier, and Theodore Schneirla [37]. At Chicago Hebb took classes with L.L. Thurstone, Wolfgang Köhler, Karl Lashley, C.J. Herrick and Nathaniel Kleitman [83] and began to think seriously about the neural control of behaviour.

In November 1934, Hebb submitted a paper, entitled “The interpretation of experimental data on neural action” for his class in Elementary Neurology (Anatomy 316), taught by C. J. Herrick [59]. In this paper, Hebb drew two diagrams of possible synaptic activity during conditioning (see Fig. 1b) and stated that: “There is as yet no understanding theoretically of the process of conditioning, and when this is understood, it may throw light on the reflex activity of the [spinal] cord, and perhaps account for the existence of some of the apparently rigidly inherited patterns. The conception of block and facilitation at the synapse postulates a process which can strengthen and perpetuate a route once formed, but none whatever to account for the establishment of the route in the first place - a most important weakness in the whole theory.” ([59], page 15). Hebb had hoped to re-write this paper for publication, but this was never completed. In a letter to Babkin (Hebb to Babkin, 6 January 1935), Hebb explained that Lashley did not favor the publication of papers which did not include experimental tests of the hypothesis proposed and concluded that “Professor Lashley did say that the paper might be re-written, so as not to claim too much, but is on the whole against publication without experimental data”.

A closer examination of this paper shows that Hebb was interested in the neural route of the reflex arc. He stated that “the conception of the reflex route [is based on] the assumption of relatively unchanging paths along which an excitation peripherally aroused is conducted” ([59] page 2). Much of this essay concerns the concept of inhibition, and he stated that: “Studies of route formation, of the reflex arc, of inhibition, facilitation or synaptic fatigue, depend on the conceptions obtained from the phenomena of the isolated nerve.” ([59], page 1). He stated that “In Pavlov’s [151] discussion of the reflex he makes no mention of specific routes; his definition of the term is essentially that whatever reaction is completely determined by natural law is a reflex” ([59], page 3). What Hebb was saying in this paper was that excitation and inhibition of reflexes must occur in specific neural routes and the patterns of activity (synaptic efficacy) in these routes of nervous activity can be stimulated or inhibited by experience. One can see Hebb struggling with the concept of how a reflex arc is formed by the combination of excitatory and inhibitory stimuli. He suggested that there was an alternative to Pavlov’s conception of the neural route of the reflex:

“In his interpretation of his data, of course, Pavlov [151] represents the most complete assumption of the linear passage of excitations along specific routes. Not even in his defence of the reflex conception and of his interpretations does he make any effort to justify the point-for-point conception of the receptor surfaces of the cortex; yet the actual data suggest, if we have not this preconception, a rather different process of excitation. The alternative, that excitation spreads at every synaptic level, is both more in conformity with the neurological facts of structure and better able to account in a simple manner for the “generalization“ of stimuli. On this assumption, it is clear, there is not a separate specific cortical excitation corresponding to each peripheral excitation, but a diffuse excitation of which a large fraction might be common to two or more peripheral excitations. This diffusity would be narrowed down to a more specific focus only with the process of differentiation.” ([59], page 7).

In preparation for rewriting this paper he added a note that references would be made to Lorente de Nó (1933 and 1934), Forbes, Herrick (1929), Uexkull, Goldstein, Graham-Brown ([59], page 17). Appended to this paper was a drawing of a possible mechanism of reflex action (see Fig. 1c).

## Unpublished notes on scientific methods in psychology [60]

While in Chicago, Hebb wrote five chapters (90 pages; of which I have found only 69 pages) of a proto-book entitled “Scientific methods in psychology: A theory of epistemology based on objective methods in psychology” [60]. In this manuscript, Hebb tried to integrate what he learned about the study of mind as a philosophy student with the neurophysiology he was learning in Chicago. In chapter I, “The theory of mind”, he discussed the philosophical concept of mind and the introspective method in Psychology. He rejected introspection as a method and then attempted to integrate the theory of mind with the concepts of Behaviorism and the physiological basis of mind. In chapter 2 “Idealism”, he considered the role of the brain and the mind in perception. While he did not get very far with his arguments about the physiological basis of mind, there are some glimpses of his thinking on mind and brain. First, he states that:

“ We can say, like primitive man, that the existence of the mind is to be inferred from human (or animal) activity; we can say, strange as it may appear to the Behaviorist, that mind is itself, and always has been, simply a theory of behavior.” ([60], page 22).

Second, he begins to develop a physiological theory of mind:

“In order to understand human activities in terms of physiological theory it is not sufficient to substitute the words ‘conditioned reflex’ for ‘association’, and ‘brain’ for ‘mind’, thinking of the brain as a distinct organ receiving impulses from different routes, each route indicating some distinct sensory perception. The modernising of older forms of thought has sometimes been rather ingenious. As soon as the neuron-synapse theory began to make it possible to think of a completely neural determination of human activity, it was assumed frequently that cortical or at any rate cerebral processes alone correlated with the process called conscious. There may be no consciousness without the cortex; but there is no reason to conclude that in the presence of the cortex the functioning of sensory and sub-cerebral neurons have nothing to do with ‘conscious’ processes.” ([60], page 39).

This proto-book includes chapters on “The induction in scientific method” (Chapter III) and “Mathematical method, probability, and deductive reasoning” (Also labeled Chapter III) and ends on page 69 without returning to a discussion of the physiological basis of behaviour. Parts of this book were to be included as a final chapter in *The Organization of Behavior* but were deleted during the writing process. Some of the ideas expressed in this manuscript, however, did find expression in *Essay on Mind*, Hebb’s final book [84]. It is important here as it shows Hebb’s early struggle to develop a physiological theory of psychological processes.

## Hebb’s PhD at Harvard [61]

Only a year after Hebb arrived in Chicago, Lashley accepted a position at Harvard and Hebb completed his PhD thesis at Harvard on the visual abilities of rats reared in the dark [62, 63]. He also completed the research that he had started in Chicago on field orientation in rats, and in these papers Hebb [64, 65] first used the term “Organization of Behavior”. In these early papers, Hebb tried to develop better behavioral tests for the study of the effects of cerebral lesions. He stated that: “It is clear that the success of the physiological analysis depends on the adequacy of the behavioral tests used. Much of the difficulty found in the evaluation of the effects of cerebral destruction, both clinically and with animal experimentation, is due to the fundamental difficulty of the analysis of behavior and to the unsatisfactory available accounts of it.” ([64], page 333).

When Hebb came to write *The Organization of Behavior*, he re-analyzed the data from his PhD experiments. He had originally reported that rats reared in total darkness learned a visual discrimination task in the same way as normally reared rats [62, 63], but when he re-examined his own data, he found that the dark-reared rats took six times as many trials as the normally reared rats to learn a discrimination of horizontal versus vertical stripes, and twice as many trials for discriminating erect versus inverted triangles ([77], page 113). In his memoirs ([83] page 286), he wrote that “It was nine years later, when I was trying to account for some of Wilder Penfield’s brain-injury results and developing the ideas that led to *The Organization of Behavior*, that I went back to my own published data and for the first time saw what kind of beast they were.” Of these papers, Hebb notes: “The results were interesting. In fact they were very interesting, but the fact also is, as far as I can discover, that no one at the time ever looked at either of my two papers [62, 63]”.

## At the Montreal Neurological Institute (MNI) with Wilder Penfield (1937–39) and Queen’s University (1939–1942)

After graduating from Harvard, Hebb moved back to Montreal to study the psychological effects of temporal lobe and frontal lobe surgery in the patients of Wilder Penfield, the founder of the Montreal Neurological Institute [66, 67]. His most complete study [88] was of patient KM, who had a frontal lobotomy. Hebb tested this patient before and after surgery and found little effect of the surgery on his scores on the standardized tests available at the time, and concluded that the removal of large amounts of frontal lobe tissue either had no effect on the mental abilities of the patient or that the tests used were not sensitive enough to detect the effects of the surgery. His experiences in testing patients at the MNI led to a number of ideas about the nature of intelligence and how it should be tested [87]. Hebb also observed that lesions of different brain areas produced different cognitive impairments and that the age at which a brain injury occurred was important in determining its effects on intelligence [68]. In this paper, he also observed that intelligence was composed of two components; a fixed component and a variable component that could be influenced by environmental experiences. He called these “Intelligence A and intelligence B” and, as has been shown elsewhere [20], Raymond B. Cattell took this idea of two types of intelligence and renamed them fluid and crystallized intelligence [29]. In *The Organization of Behavior* (page 294) Hebb used the terms Intelligence A and Intelligence B and did not refer to fluid and crystallized intelligence.

In 1939, Hebb took a position as Lecturer in Experimental Psychology in the Philosophy Department at Queens University in Kingston, Ontario. It is possible that he learned a great deal from George Humphrey, the head of his department, who had written a book entitled *The Nature of Learning* (1933), but there is only one reference to Humphrey [94] in *The Organization of Behavior*. It was at Queen’s University that he designed a variable path maze with a student, Kenneth Williams, the so-called Hebb-Williams maze [89], which he later used to test rats reared in enriched environments [76].

## At the Yerkes primate center with Lashley (1942–47)

When Karl Lashley became the director of the Yale Primate Laboratories at Orange Park, Florida in 1942, Hebb was hired as a Research Fellow. He conducted studies on fear and anger in chimpanzees and related these findings to human emotionality [73, 75]. He also conducted a study on the behaviour of dolphins [130] and continued his work on the development of rat intelligence. To determine the effects of early experience on learning, Hebb reared rats as pets at home and showed that enriched experience during development resulted in improved maze learning in adulthood. Only an abstract of this study, which was presented at an APA meeting, was published [76]. These results formed the basis of later studies at McGill on the effects of environmental enrichment on behaviour by Hebb’s students and established the field of research on how environmental influences shape neural development (see [21]).

**Fig. 2:**
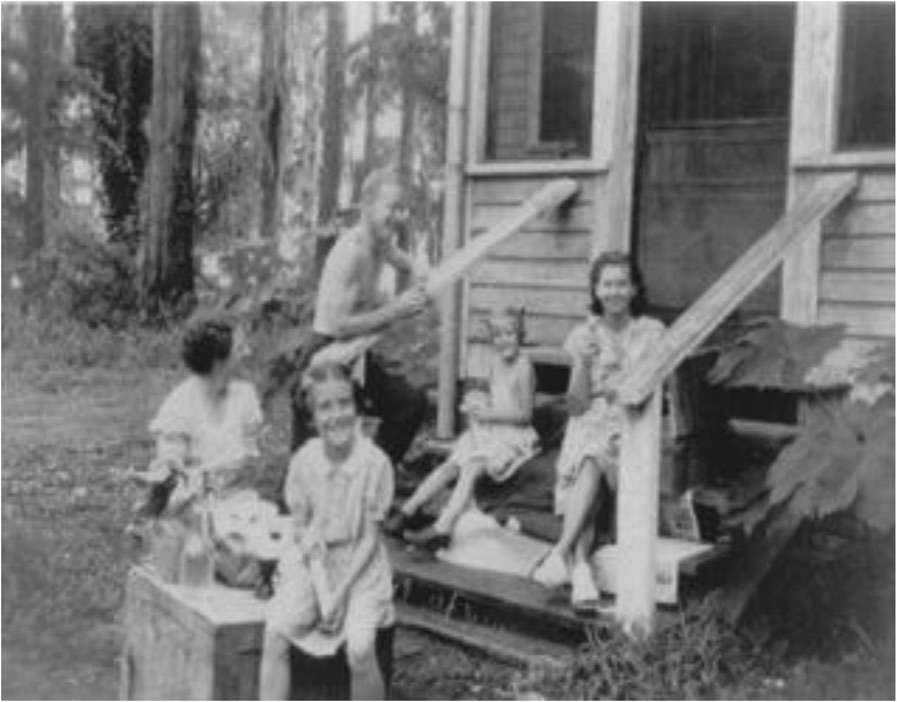
Hebb and his family in Orange Park, Florida in the 1940’s. This photo shows Hebb, his wife and two daughters and Helen Riesen. Reprinted from Dewsbury DA. Monkey farm: a history of the Yerkes Laboratories of Primate Biology, Orange Park, Florida 1930–1965. Lewisburg: Bucknell University Press; 2006 [38]. Copyright (2006), with permission from the author and from Bucknell University Press

During his years at Orange Park (Fig. 2), Hebb completed the first seven chapters of a manuscript of a book, eventually published as *The Organization of Behavior.* The intellectual climate at the Yerkes laboratories, as described by Dewsbury [38], stimulated Hebb to return to his earlier ideas on the physiology of behaviour and to formalize these in a coherent form. In the preface to *The Organization of Behavior*, Hebb states that “My greatest debt, perhaps, is to the weekly colloquium and the persistent theoretical debate at the Yerkes Laboratories of Primate Biology between 1942 and 1947; and to a small group taking part therein who have also read the entire manuscript and have contributed greatly to it -- Professor Harry W. Nissen, Mr. and Mrs. Robt. Blum and Dr. Austin Riesen.” ([77], page viii).

## Hebb’s influences in writing the organization of behavior

**Fig. 3:**
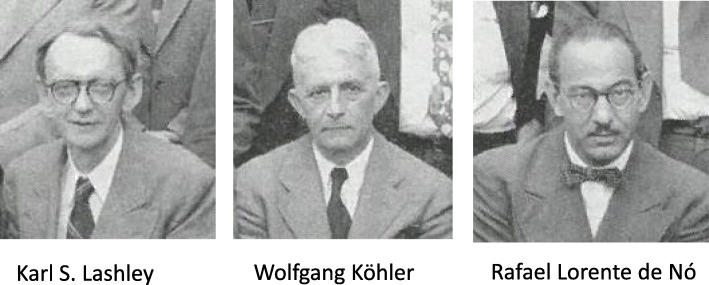
Three of the most important influences on Hebb. Karl S. Lashley, Wolfgang Köhler and Rafael Lorente de Nó in 1951. Reprinted from Jeffries, L.A. 1951. Cerebral Mechanisms in Behavior: The Hixon Symposium. New York: John Wiley and Sons. (Frontspiece). Copyright (1951), with permission from John Wiley and Sons

Hebb had a number of influences in writing *The Organization of Behavior.* These included some of the textbooks available at the time, the work of Lorente de Nó on the synapse, the work of von Senden on visual development and, as mentioned above, his colleagues at the Yerkes Primate Centre. Lashley himself was both an influence and an impediment. Figure 3 shows three of the most important influences on Hebb: Lashley, Köhler and Lorente de Nó. In the Preface to *The Organization of Behavior*, Hebb notes that there were five textbooks that he would recommend as background reading:

“Though I have done my best, it may be chimerical to hope that my discussion is extensive and clear enough to stand on its own feet, for the nonpsychological reader. The reader who needs it will find more of the details of psychological theory in Morgan [139] on physiological psychology, Hilgard and Marquis [91] on the theory of learning, Woodworth [189] on ‘experimental’ (normal human adult) psychology, and Moss [140] or Maier and Schneirla [127] on animal psychology. Of these, Morgan is most directly relevant, and in several places I have assumed a knowledge of fact to the extent provided by his text.” ([77], page vii).

In this section, I provide brief outlines of the main influences on the development of Hebb’s theories as presented in *The Organization of Behavior*. These include the textbooks of Hilgard and Marquis [91] and Morgan [139], the work of Lorente de Nó ([123, 124], and von Senden [186], Wolfgang Köhler and Gestalt Theory, and Lashley himself.

### Hilgard and Marquis [91]

Hebb gave a great deal of credit to Hilgard and Marquis [91] for stimulating his ideas on synaptic change in learning. Early in 1944 Hebb learned that Lorente de Nó [123–125] had recently shown that closed circuits were to be found throughout the brain, and “that one neuron by itself may not be able to excite a second neuron at the synapse, but can do so if supported by simultaneous action from another neuron” ([83], page 295). It was these ideas of Lorente de Nó that Hebb realized “were just what he needed to develop a neurological theory of the mind” ([134], page 127). In his book *Essay on Mind*, Hebb ([84], page 83–84) said:

“The problem of perception remained intractable for about five years (1939 to 1944) and as a result I made no progress in my attempt to understand concepts and thought. In fact, by 1944 I had given up trying to solve the problem. What happened then was that I became aware of some recent work of Lorente de Nó in conjuction with some observations of Hilgard and Marquis [91], which led me to think of a possible solution for a different problem: the problem of set and attention. And this brought me straight back to concepts and the thought process, but now from a different point of view.”

The section of Hilgard and Marquis [91] that Hebb referred to is entitled “The nature of synaptic modification” (pages 326–334) in which the ways that synaptic change can lead to the permanent neural modifications responsible for learning are outlined. Five theories were described: (1) anatomical growth of axons and dendrites; (2) changes in the physico-chemical properties of the nerve cell; (3) continued activity in closed neural chains, (4) the influence of bio-electric fields and (5) Inhibition. According to Hebb [83] it was the discussion of the work of Lorente de Nó in these pages that inspired him to develop the idea of a reverberating circuit in the cell assembly.

### Morgan [139]

**Fig. 4:**
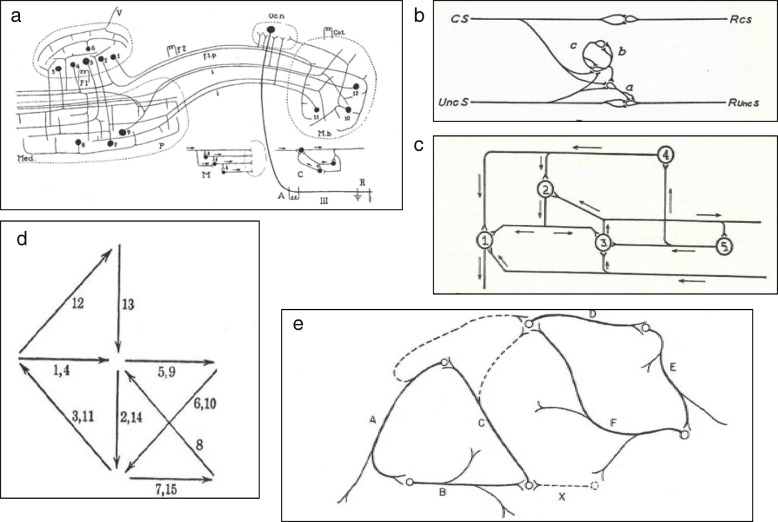
Reverberatory circuits and the cell assembly. These figures show the evolution of the reverberatory circuits that Hebb developed into his cell assembly. (**a**) A diagram of the pathways connecting interneurons among themselves and with the ocular motoneurons. V = vestibular nerve; I to 6 = cells in the primary vestibular nuclei; 7, 8, 9 = cells in the reticular formation in the medulla (Med.) and pons (P.); 10, 11, 12 = cells in the reticular nuclei in the midbrain (M.b.); Oc.n. = oculomotor nuclei; Fl, F2 and Col. = positions of the stimulating electrodes. The response of the motoneurons can be recorded with electrodes (R) from the trochlear or oculomotor nerve (III). Delivery of a shock to these nerves outside the brain stem through electrodes (A) causes the arrival of antidromic impulses at the motoneurons. The diagrams below illustrate the two types of chains, M = multiple and C = closed, that are found in the internuncial system. The closed loop system (C) represents the reverberatory circuit. ([125], page 407). (**b**) Schematic arrangement of neurons to account for conditioning by closed chains. The assumption is made that activation of a nerve cell occurs only when the cell receives excitation from two axon endings simultaneously. Impulses in CS are ineffective with respect to neurons a and b. If impulses in UncS and CS arrive simultaneously, however, they summate to excite b. This sets the closed chain b-c in continuous activity, and impulses in CS now summate with collaterals from fibers in the chain to activate a and RuncS. Simultaneous excitation of b will occur by chance if CS and UncS are stimulated together repeatedly. The chances of simultaneity are increased if the frequency of impulses in CS and UncS is greater; i.e., if the intensity of the stimuli is greater. While the speed of conditioning in any single neuron unit is thus largely a matter of chance according to this scheme, the sum of the changes in many such units would result in a gradual increase in the number of RuncS fibers activated. This scheme is not elaborated here to account for extinction, or other phenomena of conditioning. Inhibitory effects might be introduced by the addition of specific inhibitory collaterals, or by consideration of temporal relations resulting in refractory period decrement. CS = Conditioned stimulus; UncS = Unconditioned stimulus; Rcs = conditioned response; RuncS = Unconditioned response. (From [91], page 331). (**c**) Diagram of neurons and their synaptic connections illustrating the principle of recurrent (reverberatory) nerve circuits as seen in the IIIrd nerve nucleus (After Lorente de Nó). (From [139], page 64). (**d**) Hebb’s diagram of his cell assembly. Arrows represent a simple “assembly of neural pathways or open multiple chains firing according to the numbers on each (the pathway “1, 4“ fires first and fourth, and so on), illustrating the possibility of an “alternating“ reverberation which would not extinguish as readily as that in a simple closed circuit. (From Hebb [77], page 73). (**e**) An illustration of the way in which learning might modify the functioning of cortical circuits and establish a cell-assembly. It is assumed that A-B-C and D-E-F, in association cortex, are excited by the same sensory event (axons from the sensory cortex are not shown, but it is assumed that they excite these cells separately). If A then delivers impulses to B at the moment when B is being fired by axons from sensory cortex, the synapse A-B will be “strengthened”, and similarly with the other synapses. As a result of this strengthening the excitation of one cell may become able to set up reverberation in the circuit. Broken lines show possible connections between the two circuits, which would permit them to function as one system. (From [80] page 104). **a** Reprinted from Lorente de No R. Transmission of impulses through cranial motor nuclei. J Neurophysiol. 1939;2:402–64 [125]. Copyright (1939), with permission from the American Physiological Society. **b** Reprinted from Hilgard ER, Marquis DG. Conditioning and learning. New York and London: Appleton-Century Company; 1940 [91]. Copyright (1940), with permission from Appleton-Century-Crofts. **c** Reprinted from Morgan CT. Physiological Psychology. New York and London: McGraw-Hill Book Company Ltd; 1943 [139]. Copyright (1943), with permission from McGraw Hill. **d** Reprinted from Hebb DO. The organization of behavior; a neuropsychological theory. NY: Wiley; 1949. [reprinted 2002 by Lawrence Erlbaum Associates, Mahwah, New Jersey] [77] Copyright (2002), with permission from Lawrence Erlbaum Associates. **e** Reprinted from Hebb DO. A textbook of Psychology. Philadelphia and London: W. B. Saunders Company; 1958 [80]. Copyright (1958), with permission from Mary Ellen Hebb

Another reference cited by Hebb, *Physiological Psychology* ([139], pages 61–69) also has a section on “synaptic functions” which contains a diagram of Lorente de Nó’s recurrent nervous circuits and Gasser’s [46] diagram of reciprocal inhibition. Thus, the ideas of Lorente de Nó were part of the Zeitgeist as Hebb started to write his book. Figure 4 shows the different representations of Lorente de Nó’s recurrent nervous circuits. Morgan ([139], pages 137-141) also discussed spontaneous neural activity and referred to the data of Weiss [188] on tadpoles and Jasper [98] on brain waves in humans, exactly the papers used by Hebb ([77], pages 5–10). In his discussion of the neural basis of learning, Morgan ([139], pages 518–525) describes the neurobiotaxis, synaptic resistance, fiber conductance, reverberation and resonance theories as well as the drainage theory and the irradiation theory. He also includes the Gradient theory and the Pattern Theory of Lashley. Hebb ([77], pages 10–11) states that both Lashley and Köhler opposed the theory of neural connections as the basis of learning, but he notes that their critiques applied to the “older theory of linear, sensori-motor connections, in which a single cell was supposed to be always capable of exciting a single cell with which it synapsed”. This was the idea of a rigid synaptic reflex and Hebb notes that the concept of modifiable synaptic resistance eliminated this critique.

### Lorente de Nó

Hebb ([84], pages 83–88) describes how the work of Lorente de Nó [123, 124], as discussed in Hilgard and Marques [91] and Morgan [139] inspired his ideas on cell assemblies. Until the work of Lorente de Nó, it was thought that a nerve impulse was activated only by an external stimulus and lasted only a few milliseconds. Lorente de Nó showed that nerve fibers could have recurrent loops and Hilgard and Marquis ([91], page 327) suggested that “If two cells are simultaneously excited, the resulting ionization is assumed to direct the growth of axons toward the cathode region, and of dendrites toward the anode, such that a new synaptic connection is established”. Hilgard and Marquis ([91], pages 329–331) also noted that the work of Lorente de Nó [124] demonstrated that spatial summation of at least two simultaneous impulses is necessary to excite a neuron and, in their section on “Continued activity in closed neural chains” (page 330), they state that:

“Recent observations indicate that the path of neural impulses from sensory to motor nerves is not a single “straight through” pathway [124]. Histological and experimental data indicate that the internuncial neurons are arranged in two types of chains: multiple chains, in which several collaterals of a single fiber, after traversing one or more synapses, converge upon a motor neuron, and closed chains, in which a collateral excites a circle composed of several neurons. In the latter case, the chain of neurons may maintain its activity indefinitely in the absence of peripheral afferent impulses. This arrangement suggests another possible mechanism of learning which would not necessarily involve any permanent alteration in the physico-chemical properties of the neurons. Closed chains, set into activity by the training procedure and continuing in the absence of any external excitation, would summate with otherwise inadequate afferent impulses to produce the conditioned response.” (See Fig. 4b).

Hebb cited the work of Lorente de Nó ([123–126]) and all of the papers in the 1939 special issue of the *Journal of Neurophysiology* (volume 2, number 5) on the synapse. Hebb’s indebtedness to Lorente de Nó has been discussed by Martinez and Gil [128] and by Haider [50], who points out that Lorente de Nó, Hilgard and Marquis and others whose work influenced Hebb were all at Yale University in the 1940’s. In addition, Robert Yerkes who founded the Primate Research Centre in Florida did so under the auspices of Yale University [38]. Only after Lashley became the director did the Yerkes Primate Centre become a Harvard University facility, at which Hebb worked as a Harvard employee.

### von Senden

In his book *Essay on Mind*, Hebb ([84], pages 89–91) described how the work of von Senden [186] and Riesen [159] on visual development influenced his thinking during the period between 1944 and 1945 in which he was developing his theory:

“Nine years earlier in fact I had reared rats in darkness myself and tested their vision, as the basis of a Ph.D. thesis. When I recalled that work I recalled too a feeling that there was something peculiar, something remarkable, about a book I had read at the time. The book was von Senden’s [186] valuable compilation of all the published case reports of people who were born blind, with cataract, and who were later made able to see by removing the cataract. I looked at von Senden again and was astonished at what I found. His subjects were in effect blind at first. They could distinguish and respond to colors but had practically no perception of shape or pattern. A prolonged learning process was needed after operation before the patient began to see the world in the way that a normal person does” (page 90).

Hebb ([84], page 90; See also page 113 of Hebb [77]) discusses the errors in his own PhD thesis:

“My failure to see the apparent meaning of von Senden’s evidence is a perfect example of how theory, firmly entrenched, can block one’s vision. My thesis research was done under the influence of holistic and more or less nativistic ideas--with the result that I did not see the implication even of my own data. My experiments had shown no difference in the perception of brightness and size by normal and dark-reared rats. I went on to look at pattern perception and found that the perception, once established, is about the same in normal and dark-reared--patterns that look alike to one look alike to the other but failed totally to reflect on the fact that establishing the perception took six times as long in the dark reared. Only when I came back to my own published data, nine years later and with light from another theoretical idea, did I see what I had done.” (page 90).

At about the same time, Austin Riesen [159] tested the vision of two chimps reared in darkness and found absolutely no evidence of visual discrimination ability. Until the research of von Senden and Riesen, many had thought that there was an innate visual organization of the brain as Hebb [62, 63] “saw” in his PhD thesis. As Riesen ([159], page 108) states, “The prompt visual learning so characteristic of the normal adult primate is thus not an innate capacity, independent of visual experience, but requires a long apprenticeship in the use of the eyes.” Given the evidence of von Senden and Riesen’s research, Hebb ([77], Chapter 6) proposed that the young chimps reared in the dark had not formed any visual cell-assemblies ([84] page 91). In his work with fear in monkeys, Hebb [73] proposed that fear was also a disruption of cell assemblies. Von Senden’s book was not translated into English until 1960 and in this book, Riesen [160] wrote a short commentary discussing the influence of von Senden on Hebb’s ideas.

### Köhler and Gestalt Theory

Although Hebb [77] did not specifically acknowledge his debt to Wolfgang Köhler and Gestalt Theory in the Preface to *The Organization of Behavior*, Hebb had taken a course from Köhler in Chicago and was well versed in Gestalt Psychology. One might argue that Hebb’s thoughts on the failings of the Gestalt theory of perception stimulated the development of his cell assembly theory (see Section “The first draft of The Organization of Behavior” below). In his Scientific Method in Psychology, Hebb ([60], page 36–37) is critical of Köhler and in his Precis ([71], page 6 and page 20), Hebb criticises the approach of the Gestalt psychologists to perceptual learning. He said “In general, the Gestalt treatment of the figure-ground relationship has excluded the case in which experience is of primary importance, although not denying its existence” ([71] page 6). Following the introductory chapter, Hebb ([77], page 17) begins *The Organization of Behavior* with “a revision of perceptual theory” and points out two faults with Gestalt theory: (1) that perceptions depend “on a pattern of excitation whose locus is unimportant”; and (2) that when one perceives a simple figure “one perceives it directly as a distinctive whole, without need of any learning process and not through prior recognition of the several parts of the figure”. The focus of the cell assembly and phase sequence theory in the first chapters of *The Organization of Behavior* is on perception, and it is interesting that Hebb ([77], page 17) says in a footnote to Chapter 2, that “This and the following chapter may be disregarded by the reader who is not particularly interested in the theory of perception”. However, the chapters that define the cell assembly and phase sequence (Chapters 4 and 5) are entitled “the first stage of perception” and “perception of a complex” and focus on perceptual learning. It is not until Chapter 6 that Hebb begins to talk about learning in general.

Although Hebb did not refer the reader to the works of Köhler [104] or Koffka [103] in the Preface to *The Organization of Behavior*, these were important for his PhD research [62, 63]. In his PhD thesis he stated that “The Gestalt school ... implies the innateness of certain organizations of the visual field in stressing the dominance of primitive Gestalten over learned ones. The kind of evidence cited here includes the persistence of visual illusions in spite of experience, and the failure of experimental subjects to pick out figures which they have been trained to recognize, when these are part of a more dominant figure” ([61], page 2). Once Hebb understood the data of von Senden and reinterpreted the data from his PhD thesis, he understood the flaws in the Gestalt theory and this stimulated his “revision of perceptual theory”, which was to describe (1) the neural pattern of perceptions and (2) how these were learned by experience.

### Karl Lashley

Much has been written about the relationship between Lashley and Hebb [24, 144, 146] and, although it could be argued that everything Hebb learned about the brain, he learned from Lashley, he did not always agree with Lashley. It was Lashley who proposed Hebb’s PhD dissertation topic, based on his tests of the innate organization of vision [120] and it was Lashley who stimulated his experiments on the effects of cortical lesions on spatial learning [64, 65]. Indeed, the majority of Hebb’s published papers have references to Lashley’s work. In chapter 3 of *The Organization of Behavior*, Hebb says that Köhler and Lashley are the only writers who recognize “the real problem of the neural mechanisms of perceptual integration and attempt an adequate solution” (page 38), but he was not happy with their solutions and suggested that “the line of thought that they have chosen may be a blind alley”. Hebb’s theory was a response to the theories of Köhler and Lashley, but the development of this new theory depended on his being in Lashley’s lab environment in Chicago, at Harvard and in Florida. The development of Hebb’s theory also depended on other students in Lashley’s lab, especially Frank Beach, whose paper on the neural basis of sexual excitement provided Hebb with the concept of the central excitatory mechanism [11] for his study of fear in chimpanzees [73]. This paper introduced the concept of the “phase sequence” and the dual process based on the separation of external stimuli and autonomous central processes, which were discussed in detail by Beach [11]. As noted below, Hebb felt so strongly about his debt to Lashley that he asked him to be a co-author on *The Organization of Behavior*.

## Hebb’s notes for *The Organization of Behavior*

Although he does not mention his unpublished notes in either of his autobiographical papers [81, 83], these show how he developed the ideas for *The Organization of Behavior*. Hebb [69] first outlined his ideas in five pages of typed notes, which contain his early thoughts on the term “organization”. In this definition, he considered both behavioral and physiological organization and considered the concept of “set”, which he used to denote the pre-existing state of the nervous system encountered by an incoming stimulus. A key concept in these notes is the LNC, which was the ‘Lorente de Nó circuit’. Hebb ([69], page 1) takes this as his central concept in the organization of behavior:

“What I mean by organization behaviorally: ready identification or discrimination, ease of recall and accuracy of recall or identification, ease of association; recognition in any circumstances. And by physiologically: assumption of need of number of parallel LNCs for stable response or perception, possibly of good organization by temporary facilitations, possibly of good organization by innate physiological and anatomical factors, possibly of organization by structural modifications.”

In the spring of 1945 Hebb [70] made four pages of hand-written notes in which he worked out his theory of the ACA = Autonomous Central Action (also ACP = Autonomous Central Process). Hebb ([70], page 1) stated: “That ACA must be considered the direct determinant of behavior; that ACA events are a joint function of afferent activity and preceding ACA activity (influence of ACA on perceptions, also, was however seen only after system began to be developed).” A third set of notes for *The Organization of Behavior* was typed between March and July 1945. In these notes, Hebb ([71], page 1) states that:

“the attempt is made to show that a schema of the physiological control of behavior can be developed by utilizing these preliminary ideas. The schema does not make specific predictions, and thus suggests only a reformulation of the problem of the control of behavior, instead of providing an explanation. There is an approach to explanation, however, in the fact that a variety of psychological problems may possibly be unified, and how it is possible for example to conceive of a mechanism of the effect of attention on learning, in physiological terms; and suggests that if the hurdle of perceptual generalization can be got over, the problem of motor generalization and its perceptual control becomes physiologically explicable (in general terms, of course, and for simple cases).”

These notes include a rough draft of Fig. 3 on page 52 of *The Organization of Behavior*. The second section of these notes entitled “A schema of perception” [72] contains a draft of Figure 11 in *The Organization of Behavior*. These four sets of notes [69–72] provide an overview of Hebb’s theories that were presented in the first two chapters of *The Organization of Behavior*. Many of Hebb’s notes refer to KSL (Lashley) and in June 1944, he noted that he was “prepared to accept - even welcome-innate organization such as Dr. Lashley has suggested”, but by the Precis of 1945 [71], he has re-discovered von Senden and wrote that “Senden’s [186] evidence is that both the normal associability and the normal generalization of simple figures such as circle, triangle and square are built up by experience” ([71], page 9).

In his paper “on the nature of fear” Hebb [73] outlined some of the ideas that he was introducing into his book in progress. These include discussions of central versus sensory factors in determining fear and the inference of a “central excitatory mechanism or central motive state” ([73], page 267), which had been presented by Beach [11]. He described temporal patterns of cellular activity as a ‘phase’ and stated that “Behavior is directly correlated with a phase sequence which is temporally organized” and emotional activity disrupts the phase sequence ([73], page 269). He said that “Subjectively, the phase sequence would be identified with the train of thought and perception”. Indeed, the concept of the phase sequence is spelled out very clearly in this paper, which is the immediate precursor to *The Organization of Behavior*. However, the concept of the cell assembly had not been developed and Hebb ([73], page 269) used the term ‘phase’ for “a specific pattern of cellular activity”.

## The first draft of *The Organization of Behavior*

A draft of *The Organization of Behavior* was finished in 1946 and given to Hebb’s colleagues at the Yerkes Primate Centre to read. As noted on the typed manuscript [74] these included Robert and Josephine Blum, George Clark, Henry Nissen, Roger Sperry, Austin Riessen, Karl Lashley, and Karl Pribram. Dewsbury ([38], pages 174–176) gives the details of the staff members of the Yerkes labs during the years that Lashley was the director. Hebb also sent copies to Frank Beach, Edwin Boring at Harvard, Professor R. B MacLeod at McGill, Dr. J. C. R. Licklider, Dr. J. G. Beebe-Center and Dr. G. A. Miller, as well as others. However this draft contained only chapters 1 to 7 of the published version of *The Organization of Behavior*. Chapters 8 to 11 were added after Hebb returned to McGill University in the autumn of 1947.

The draft of *The Organization of Behavior* that was submitted in 1946 was different in a number of ways from that which was finally published in 1949. The published book has a preface and an introduction, while the 1946 manuscript begins with chapter 1. Sub-titles of chapter sections also differ. For example, the section “Rejecting the assumption of a complete sensory control” ([77], page 3) was “Autonomous central process versus sensory dominance” in the 1946 draft. The last section of Chapter 1, “The mode of attack” has been severely trimmed in the book. In the original manuscript, Hebb basically says that Lashley was wrong, Köhler was wrong, Pavlov and Hull were wrong and Skinner was wrong, and he will correct them. It is worth citing these sections in full to get the flavour of Hebb’s original mission as they were deleted when the book was published.

“If one assumes that Köhler and Lashley have provided the most nearly satisfactory account of behavior, the next step obviously is to find out what is not satisfactory about their theories, and what changes are needed.”

“The changes lead directly toward the position taken by Hull and others who have emphasized slow-increment learning as the fundamental psychological phenomenon.”

“But Hull’s formal system must also be rejected, just as Köhler’s must. Hull’s is the most consistent theory, internally, of those current today; but its consistence seems possible only by neglecting certain aspects of behavior (putting them aside for treatment later, presumably); and it makes no real attempt to define its variables physiologically. I propose to show, at the end of this monograph, that psychological theory is essentially concerned with physiological facts and concepts Skinner [173] to the contrary notwithstanding. Assuming that, and assuming that perceptual generalization must be accounted for, the work of the Gestalt school and Lashley is the only solid starting point for theory.

“Now let us see, in the next two chapters, what it is in Köhler’s and Lashley’s treatment of perception that needs revision.” ([74], page 20).

Another section which was deleted concerns Hebb’s critique of Pavlov. He says:

“Here the concepts of irradiation and concentration, as an explanation of generalization, break down. When one point on the skin is conditioned, so that its stimulation is followed by secretion of saliva in Pavlov’s method, neighboring points will also produce some secretion. This Pavlov accounts for by the notion of “irradiation” or excitation over the cortex. But with differences of intensity, instead of locus, the matter is different: now the two stimuli are projected to the same cortical point. Irradiation cannot account for the discrimination of relative intensities as it perhaps might for discrimination of relative place. Pavlov [151] failed to answer this criticism and the subsequent attempts to modify his ideas to cover it by a verbally postulated “generalization gradient” ([93, 175]) have provided no intelligible conception of a physiological mechanism.” ([74], page 44a.).

### The lattice and the schema became the cell assembly and the phase sequence

**Fig. 5:**
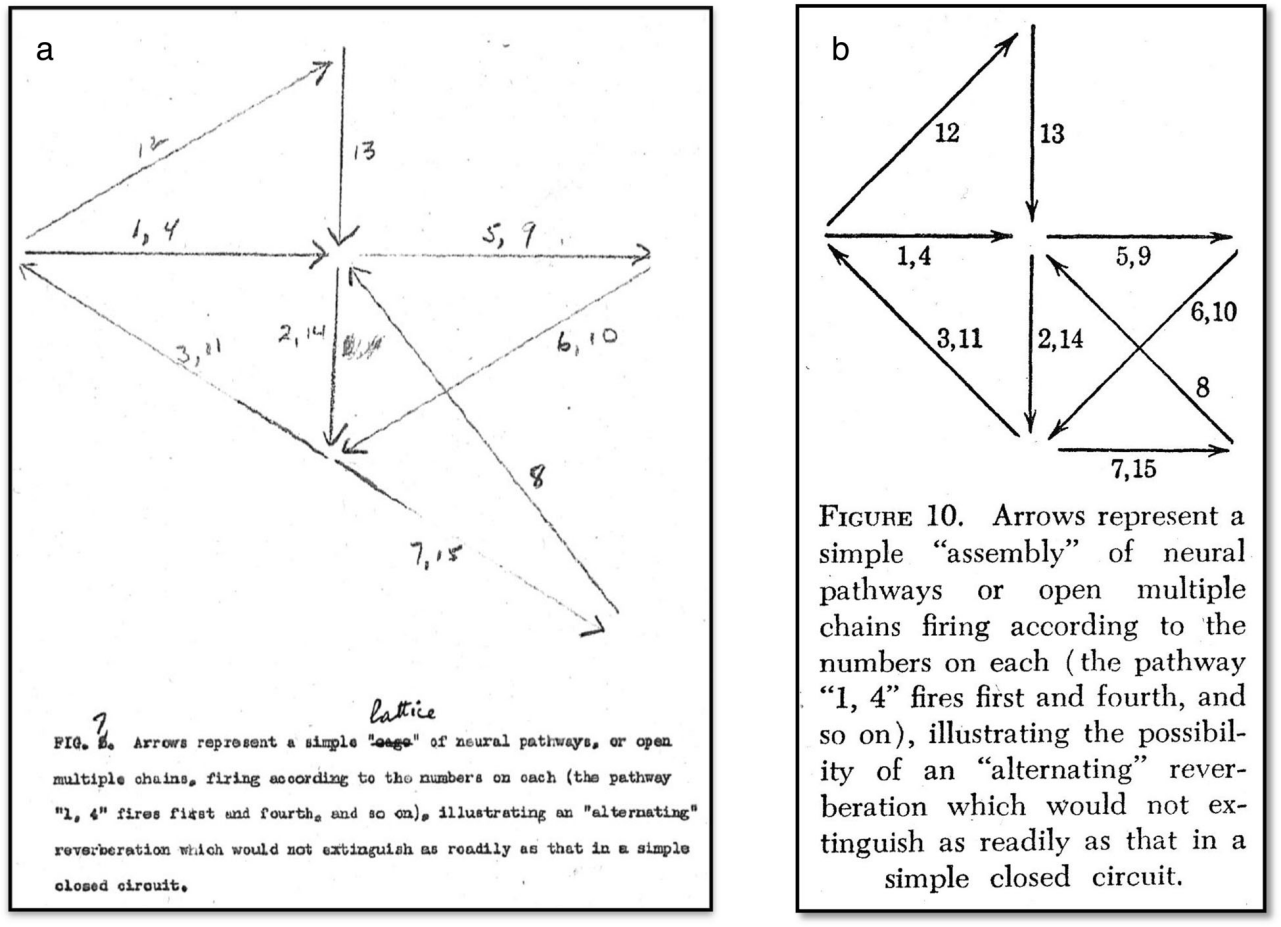
A comparison of (**a**) the original drawing of a cell assembly from Hebb’s [74] typescript in which the cell assembly was first called a cage, and then changed to a lattice and (**b**) the published figure of a cell assembly from *The Organization of Behavior* [77], page 73. **a** Reprinted from an unpublished manuscript by Hebb, D. O. 1946, entitled "Carbon of most of the original MS of my book The Organization of Behavior (while the term "lattice" was still used instead of "cell assembly" [74]. The original is held in McGill University Archives, Montreal, Quebec, file MG1045. Reprinted with permission from Mary Ellen Hebb. **b** Reprinted from Hebb DO. The organization of behavior; a neuropsychological theory. NY: Wiley; 1949. [reprinted 2002 by Lawrence Erlbaum Associates, Mahwah, New Jersey]. Copyright (2002), with permission from Lawrence Erlbaum Associates

In Hebb’s 1945 Precis [71] and in the original 1946 typescript of *The Organization of Behavior* [74] , Hebb did not use the terms “cell assembly” or “phase sequence”. In the typescript ([74], page 69), the cell assembly was called a ‘lattice’, and the phrase “the specificity of such a lattice” was changed to “the specificity of such an assembly of cells” when the book was published ([77], page 72). Figure 5 shows the original drawing of the lattice. In the original typescript there was no “phase sequence”. The term “schema” was used and Chapter 5 (“Perception of a complex: the phase sequence”) was originally entitled “Schema: the perception of a complex”. The term “phase sequence” was introduced in Hebb's paper on the nature of fear [73].

While the term “lattice” remains in the published book in only two places, (pages 72 and 75), the term “schema” is used quite often. It appears that the term schema originally referred to both the cell assembly and the phase sequence. The idea of a ‘schema’ was first used by Head and Holmes [54] to refer to a cortical representation of body posture that was built up over time. “Schemata” were defined as “organized models of ourselves” produced by incoming sensory impulses; destruction of such schemata by a cortical lesion “renders impossible all recognition of posture or of the locality of a stimulated spot in the affected part of the body” ([54], page 189). Bartlett ([9], chapter 10) used Head’s concept of schemata to develop his theory of remembering, but other than mentioning nerves, this was a cognitive, not a neurophysiological theory. He stated (page 214) that his theory “merely jumbles together innumerable traces and calls them ‘schemata’ and then it picks out a few and calls them images”. It was Hebb’s job to turn these schema into a neurophysiological concept, the phase sequence, but this was a three stage process: synaptic change leading to a cell assembly and a series of cell assemblies combining to form a phase sequence or schema. In recent studies of “memory schema” Hebb is seldom mentioned [47] but his concept of the cell assembly links Bartlett’s concept of schemata and Tolman’s concept of cognitive maps with the later theories of neural networks and “place cell assemblies” [7, 110].

### Why did Hebb change the names of the lattice and schema?

In a letter to Henry Nissen (12 September 1948) Hebb wrote that “I am now about ready to send off the MS [manuscript], with one major headache. Licklider at Harvard, who read the first half last summer, has decided strongly that “lattice” has too many wrong implications. I agreed too and must now choose another term - have you any ideas? A structural analogy seems impossible, no good term available, so I think now of some Greek word meaning a working together, or calling together, or something of the kind - if you have any ideas, shell out!” Will consult Classics Dept., try to find something innocuous and euphonious”.

On 14 September 1948, Nissen wrote back that “As to “lattice”, my impression in brief is that (1) it has some wrong implications, (2) any other term you choose will have wrong implications, (3) you will find no term that is less objectionable. Look at it this way: a lattice is an organization, an organization inferred or postulated to exist in neural structures. So, since you incline towards the Greek, “neuromorphon” or “neuro-organon”. If I have any better brainstorms, I’ll let you know.” On 22 September 1948, Hebb wrote to Nissen that “I have changed the term “lattice” to “cell-assembly”. This at first glance sounds quite peculiar; but I think you may feel, after you have seen the way it works out in the actual context of the discussion, that it may be a very happy solution with fewer drawbacks than most of the others”. Today we should applaud Hebb’s choice of the cell assembly; it could have been a neuromorphon or neuro-organon.

In this context, it is interesting that Lashley [117] discussed the organization of the nervous system in terms of schema and lattices. Lashley had read Hebb’s manuscript in 1946 and commented upon it in Feb 1947, but this paper does not mention that manuscript, nor does it mention Bartlett [9]. The influence of Hebb might be felt in the statements that “ anatomic studies suggest that a network or lattice of nerve cells forms the basis of the central integrative processes” (page 36) and the sentence that: “I shall designate these patterns of interacting cells as neural schemata” (page 36). However, unlike Hebb, Lashley included the concept of neural inhibition in his diagrams.

## Commentaries on the first draft

Many people wrote comments on the early draft of the manuscript, including Lashley, and Boring.

### Boring

Boring [16] appears to have read the first 104 pages (4 chapters) and wrote to Hebb that he should be briefer, less defensive, more positive, and more kindly, gay and friendly. Boring sent 6 typed pages of comments to Hebb with a page-by-page critique. It appears that this critique was based on an earlier version of the book than the Hebb 1946 typescript that I have [74]. However, one comment on Chapter 3 is worth noting. In his comments, Boring says “ This is a good chapter and important. These things need to be said, though it is plain that their chief importance is as an introduction to something else that I have not come to. I think you [r] criticism of K and L [Köhler and Lashley] is valid and proper and courteous. You seem to be harder on K than on L, but then I think K is much wronger than L. So far there is no reason at all to hesitate about publishing these comments on Lashley. They do not sound disloyal. They seem rather to be supporting him toward what he has always been after, even though disagreeing in particulars. In this way Stevens and I keep supporting each other and referring to each other, while dissenting from each in minor details.” (Boring to Hebb [16], page 5).

### Lashley

In February 1947, Lashley sent Hebb two pages of comments on the first 91 pages of the manuscript. A few of Lashley’s comments might be noted. “P.13. Statements in 2 following Pp [paragraphs] are questionable without amplification. Neither spontaneous activity nor rev. [reverberatory] circuits in themselves provide solution of the problems of the synaptic theory of learning.” “P. 53. configuration theory does not account for anything - it sets problems. Köhler.” Hebb’s notes on these two pages say “These 2 sheets are Lashley’s commentary (he didn’t approve) plus all entries in red on the MS itself.”

### Hebb asks Lashley to be a co-author

As he wrote his book, Hebb realized how indebted he was to Lashley. In his autobiographical paper, Hebb said that “I went to Lashley and proposed that we work it out [the concept of a schema] and publish together, since this would have to be a book-length job, which I was not ready to face by myself. Also I hoped that he could devise a better treatment of perception (I thought this part of the schema was wrong, but could find no alternative). Between us, a much better job was possible, in both theory and presentation; and with him as joint author, the book would get a hearing that I did not expect I alone would get. But Lashley was entirely uninterested and remained skeptical.” ([83] page 296). In an unpublished autobiographical paper Hebb said “When it came to working out my theory in 1945 I could see that it was a book-length job which I didn’t feel able to tackle. I went to Lashley to ask him to be a joint author. He was quite uninterested. Together we might have done it in two years. Me it took five, and I finished it at McGill after leaving Florida” ([85], page 13). In a letter to Henry Nissen (Hebb to Nissen, 19 May 1948) Hebb says that Lashley “told me you know that the whole thing was very weak, with no value because it was so vague, which I think had a great deal to do with my patting myself on the back and being so aggressive in discussing other work.”

## Completing the Organization of Behavior at McGill (1947–1949)

In December 1946, Edwin G. Boring, the Chairman of the Psychology Department at Harvard, invited Hebb to teach a graduate seminar during the summer session of 1947 and Hebb used a draft of his book in this seminar. One of the students in this class was Mark Rosenzweig, who wrote that:

“I took a graduate seminar with Donald O. Hebb at Harvard in the summer of 1947 where the text was a mimeographed version of Hebb’s influential book *The organization of behavior* which appeared in print in 1949. Hebb’s creative suggestions revitalized theorizing and research on learning and memory, and I benefitted directly from them and from further contacts with him” [162].

When the summer at Harvard was over, Hebb moved back to Montreal as Professor of Psychology at McGill University, where he developed a graduate program in physiological psychology and completed the final chapters of his book. In the Preface to the *Organization of Behavior*, Hebb wrote:“ It is a pleasure to record my indebtedness to the colleagues who have read and improved the contents of this book. I owe much to students in a seminar at Harvard University in the summer of 1947, and in another at McGill University in the following winter”. Peter Milner, Brenda Milner and others had read mimeographed copies of the book at McGill. Brenda Milner ([132], pages 282–283) said that:

“during [Hebb’s] first seminar, we discussed this book chapter by chapter and did the relevant background reading, which covered Lorente de Nó, Marshall and Talbot, Hilgard, Lashley, and Sperry. The graduate students in this seminar included Mortimer Mishkin, Lila Ghent (Braine), Herb Lansdell, and Woodburn Heron, and discussion after the seminars often continued late into the night. It was an exciting time and hastened my decision to do a PhD at McGill. In 1949, I persuaded him to accept me as a graduate student. I wrote to Peter [Milner] enthusiastically about the Hebb seminar, with the result that he decided on a career change.”

Peter Milner ([138], page 36) said that “When I read the chapter on the cell assembly in a manuscript of *The Organization of Behavior*, I thought Hebb might be on the way to an answer. If I studied with him, I might even help him find it.” Peter Milner [133] published a revision of the cell assembly, and other papers culminating in his book *The Autonomous Brain* [135].

## Henry Nissen edits the Organization of Behavior

In June 1947, Hebb’s book was submitted to Charles Thomas publishers and it was sent to Henry Nissen at the Yerkes laboratories in Orange Park, Florida to review. It was 150 pages long and insured for $300.00 (Nissen to Hebb, letter of 21 June 1947). Nissen edited this manuscript from June 1947 to September 1948, sending Hebb numerous comments and suggestions for changes (Nissen to Hebb 11 May 1948). Nissen sent pages of typed notes on each chapter as well as “comments in pencil in the margins”. His over-all comments were that “I agree whole-heartedly with your plan to ‘tone down the criticism’ and to refrain from patting yourself on the back quite so often. A number of my criticisms would be met by those two changes. A ‘less argumentative tone’ would be an improvement”.

## Nissen turns down co-authorship

On 19 May 1948, Hebb wrote to Nissen and asked “is there any way in which I could induce you to share authorship with me, change the matter and style where you think it should be changed, developing your own ideas concerning perception and learning and motivation, perhaps writing a chapter on the relation of animal work to human problems incorporating the ideas that you probably had to skimp on in writing your handbook chapter?” (Hebb to Nissen, 19 May 1948).

Nissen replied that “I am quite overwhelmed by the suggestion that I share authorship of the book with you. A more complimentary thing has never happened to me. A quick analysis indicated several factors which may have contributed in bringing forth this suggestion: (1) An over-reaction to KSL’s criticisms - an “over-reaction“ regardless of how severe those criticisms may have been. (2) A failure on your part to realize how good the completed parts of the book are right now. Perfect? No. But it will never be perfect no matter how many collaborators you ring in on the job.” [Nissen to Hebb, 23 May 1948, concluded 29 May].

In his letter to Henry Nissen on 19 May 1948, in which he invited Nissen to be a co-author, Hebb outlined his completion of the book. He said that “What I’d like to do, therefore, is get your help re-writing, your additions and modifications of the theory. I would then write two chapters, one on emotion [chapters 8 and 9] and clinical deviations of emotion [chapter 10] as far as they touch on theory, and one on human intelligence [Chapter 11]”. In Nissen’s letter to Hebb [23 May 1948, concluded on 29 May] Nissen gave Hebb a number of bits of advice about finishing the book “within the next 8 to 12 months” and suggested that “something could be added (preferably near the beginning) which would summarize the inadequacies of conditioning theory and the essence of your form of association theory by which those inadequacies are repaired.”

In his reply to Nissen [1 June 1948] Hebb said that “I have now been through your criticisms in detail, and mean to accept practically all of the changes you suggest.” Nissen sent Hebb his critique of Chapter 8 on 13 July 1948 and Hebb sent chapter 9 to Nissen on 19 June 1948. Nissen sent Hebb his comments on the revised Introduction and on Chapter 9 on 6 August 1948, his comments on Chapter 10 on 8 September 1948 and those on Chapter 11 on 15 September 1948.

## Rejection and acceptance

On 22 September, 1948, Hebb sent the final draft of the manuscript to the publishers, Charles C. Thomas. The original title of the book was *On Thought and Behavior.* However, in January 1949, Thomas returned the manuscript, saying that they could not publish it for another year or more [letter of Hebb to Nissen, 17 Feb 1949). In a letter to Frank Beach [6 February 1949] Hebb says “Thomas has just sent back my MS, regretting he is unable to publish it on account of circumstances beyond his control - to wit, strikes and slow-downs by printers, piling up work for the past two and a half years.” Both Frank Beach and Henry Nissen wrote to the publishers John Wiley and Sons and Appleton-Century-Crofts to see if they would publish the book, and on 18 March 1949, James Helming of John Wiley and Sons wrote to Hebb agreeing to publish his book with only ‘perfunctory’ editing. In the letter, he noted that his editor-in-chief had said ‘This is by far the best-written manuscript that has come my way in some time. The exposition is lucid, persuasive, and also lively - the author need have no misgivings about the propriety of his humerous touches; they are distinctly refreshing. Unlike most factual manuscripts this one has a definite appeal on literary merits alone.’ And so it was published in September 1949 by Wiley under the title *The Organization of Behavior.*

When published, *The Organization of Behavior* was reviewed by Kuhn [107], who said that “this book will probably come to be regarded as a landmark in psychological theory.” Attneave [6] said that “I believe *The Organization of Behavior* to be the most important contribution to psychological theory in recent years.” Leeper [121] stated that “There are so many respects in which Hebb’s book is so high in quality and is so delightfully written that it will have an assured status in psychology.” Hebb’s book lived up to the reviewer’s predictions and became one of the most important contributions to psychology in the twentieth century. During the 1950’s, Hebb’s ideas found a place in many texts. Allport [3] devoted an entire chapter (entitled “The association approach, cell assembly and phase sequence”) to a discussion of Hebb’s ideas on perception. Hilgard [90] added a new section on Hebb’s neuropsychological model to the revised edition of his book and in 1959, Koch’s monumental seven volume survey of psychology as a science had a chapter by Hebb [81] and references to Hebb’s book in every volume [102].

## Hebb’s evaluation of the Organization of Behavior 10 years later

In discussing his book ten years later, Hebb ([81], p 638) said that his idea was to “deal with set and attention and perceptual generalization and learning in one theoretical framework, not have one approach for thinking, another for learning, and a third for perception -- the position in which the members of the Gestalt group found themselves.”

He went on to say that:

“My theory is the only one that attempts this, and in my opinion, to be quite frank, is consequently the only realistic attempt to deal theoretically with the problems of behavior. Skinner of course has avoided theory; Tolman and Guthrie have proposed approaches to the problem of constructing a theory, but both have remained, essentially, programmatic. Hull’s is the only real alternative to mine; and the course of development of his ideas, from 1937 to 1951, has shown a narrowing of the range of phenomena dealt with, an increasingly clear set of difficulties to be encountered even in the narrow range with which his theory does deal, and an increasing concern with minor modifications of postulates as defensive measures to meet the attacks of critics. ..... Mine, in short, is the only attempt to deal with the thought process and perception in the framework of a theory of learning. It has serious defects, but no real competitor. This fact I see as the major “evidence for the system”, together with the body of research that it has, directly or indirectly, stimulated. ([81] page 638–639).

Hebb’s theories generated a great deal of research by his students at McGill University. However, Hebb said that he “discouraged experiments designed as a test of my theory in a narrow sense, feeling that this would limit the student’s research too much.” ([81], page 637). But he did describe how the research work in his laboratory was related to the idea of the cell assembly. “The connection of my theory to some of the work is fairly clear: the studies of visual perception of Mishkin and Forgays, Orbach, Heron, and Hunton; the effects of perceptual isolation by Bexton, Heron, Scott and Doane; the role of the infant environment in mental development by Hymovitch, Forgays and Forgays, Clarke et al, Thompson and Heron, Melzack and Mahut; and the reexaminatioin of the mass-action and equipotentiality conceptions by Lansdale and Smith.” ([81], page 637).

Hebb ([81], page 639) also criticized his theory for its vagueness and the difficulties in conceiving of how the cell assembly could be subjected to experimental analysis. He pointed out that new physiological data in the decade since his book was published “would have greatly affected the formulations of the theory”. In his later papers, Hebb extended his theory to account for some of these new developments. His *Textbook of Psychology* [80] was, in many ways, a continuation of *The Organization of Behavior* and in his Presidential Address to Division 3 of American Psychological Association Hebb [79] examined the relationship of the arousal theories of Moruzzi and Magoun to his theory of motivation and described the relationship between arousal level and optimal performance as a theory for understanding motivation. Still later, Hebb ([82], page 314) suggested that inhibitory circuits were important for the functioning of the cell assembly because inhibitory circuits “serve the purpose of promptly shutting off a cell assembly once it has performed its function of firing, or taking part in firing, another.”

Hebb ([83], page 301) noted that he was surprised by the success of his book. He said that: “I expected that years of research with graduate students would be needed to get a hearing for the book once it did appear, and its immediate success was quite astonishing.” He pointed out that the arousal system was discovered by Moruzzi and Magoun in the year that *The Organization of Behavior* was published and a few years later Eccles established the existence of inhibition “which I had been afraid to postulate”. One might consider Hebb's 1980 book *Essay on Mind* [84], as a review of his thoughts on the cell assembly theory and its importance in psychology and neuroscience.

## Eight issues arising from the Organization of Behavior

The three main ideas in *The Organization of Behavior* were (1) the “Hebb synapse”, (2) the cell assembly and (3) the phase sequence. An important concept underlying these ideas was (4) the autonomous central process. Two important omissions were (5) the concept of inhibition and (6) any reference to chemical neurotransmitters. Finally, one of the reviewers of this paper asked about (7) Hebb’s ideas on the relationship between instinct and learning and (8) the relationship between the cell assembly and the engram. Each of these issues is discussed here.

### The Hebb synapse

Much has been written about the Hebb synapse [32, 105, 109, 137, 144, 166, 168, 174, 176]. The idea of the ‘Hebb synapse’ was not new to Hebb but had been thought of by many others (see [137]). As noted by Hebb ([77] page 70), “The general idea is an old one, that any two cells or systems of cells that are repeatedly active at the same time will tend to become ‘associated’ so that activity in one facilitates activity in the other.” Indeed, in his 1903 speech in Madrid, Pavlov [150] speculated on the neural mechanism underlying the conditioned reflex and presented a theory that sounds very similar to the familiar “Hebb synapse”. He stated that when the “psychical” stimuli associated with the food:

“become connected with the same nervous centre of the salivary glands to which the stimulation emanating from the essential properties of the object is conducted through a fixed centripetal path. It can be assumed in this case that the salivary centre acts in the central nervous system as a point of attraction for stimuli coming from other sensory surfaces. Thus a certain path is opened from the other excited areas of the body to the salivary centre. But this connection of the centre with accidental points is very fragile and tends to disappear of itself. Constant repetition of simultaneous stimulation by means of the essential and unessential properties of the object is required to make this connection increasingly durable.” (page 163).

Hilgard and Marquis ([91], pages 326–335) discussed the theories of synaptic function then current, as did Morgan ([139], pages 520–525). These theories included ‘Neurobiotaxis’, the formation of new synapses; ‘Synaptic resistance’, the reduction of synaptic resistance during learning; ‘Fiber conductance’, the theory that repeated passage of an impulse along an axon increased its conductivity; ‘Reverberation’, the activation of reverberatory nerve circuits; and ‘Resonance’, during learning, neurons that are out of tune, become more in tune with each other. Berlucchi and Buchtel [12] summarize a number of other theories of synaptic change underlying learning and they reprinted a letter from Hebb, written on 25 August 1977, in which he stated: “In my view, in 1946 or so when I was drafting O of B [*Organization of Behavior*], I wasn’t proposing anything new. All I considered I was doing was making a more operational statement of a widely held idea that “synaptic resistance“ was reduced whenever an impulse “crossed the synapse”.” Hebb went on to say that: “The bottom two paragraphs of page 60 (O of B) specifically make the point that it was an old idea, repeatedly rejected by critics of learning theory, but now revived in a new physiological context (i.e., the idea was not new).”

One of the main critics of learning theory at this time was Lashley, who thoroughly rejected the idea that synaptic change underlied learning. This was clear in the first sentence of his 1924 paper in which he said that: “Among the many unsubstantiated beliefs concerning the physiology of the learning process none is more widely prevalent than the doctrine that the passage of a neural impulse through the synapse somehow reduces synaptic resistance and leads to the fixation of a new habit” ([111] page 369). Lashley repeated his critique of synaptic resistance theory in his book on *Brain mechanisms in intelligence* ([112], pages 125–127) where he said that “it is not clear that the synapse is either essential or important for learning”. Lashley ([115], pages 488–494) reviewed five theories of the neurobiological mechanisms of learning and concluded that “If our analysis of the problems of behavior and of neural mechanism is correct, it follows that the current theories of neural organization have started from false premises and offer no hope of a solution of the problems”.

As pointed out earlier, Hebb had drawn out his ideas of synaptic changes in learning in his 1932 MA thesis [58] and in his 1934 essay [59]. In this essay, Hebb suggested synaptic mechanisms for both excitation and inhibition (see Fig. 1c). It is little wonder then, that Lashley harshly critiqued Hebb’s 1934 essay [59] on the neural basis of learning, as it proposed synaptic change as the neurophysiological mechanism underlying learning. And again, it is no surprise that Lashley did not want to be the co-author of a book whose fundamental premise was that synaptic change was the basis of learning and perception. Hebb was amused that as a result of his neurophysiological postulate, synaptic changes in learning had acquired his name, “because this postulate is one of the few aspects of the theory he did not consider completely original. Something like it had been proposed by many psychologists before him, including Freud in his early years as a neurobiologist.” ([134], page 127). It has been suggested that in addition to anticipating a Hebb-like synapse, Freud also anticipated the discovery of LTP [31, 165]. At about the same time as Hebb, Konorski [106] published his theory of synaptic plasticity underlying learning [13].

### The cell assembly

The concept of the cell assembly was Hebb’s greatest contribution to psychological theory according to Milner [134]. Hebb developed the cell assembly from Lorente de Nó’s reverberatory circuits and credits Hilgard and Marquis [91] for introducing him to the papers of Lorente de Nó ([77], page 61, [84], page 84). The cell assembly is thus the extension of Lorente de Nó’s [124, 125], reverberatory circuit and the “closed chain of neurons”. The cell assembly is a group of neurons arranged as a set of closed pathways ([80], page 103) that have become connected with each other by the process of perceptual learning. The self-exciting closed loops among the neurons in a cell assembly can maintain their activity for some period of time after the cessation of an external stimulus. Because cell assemblies may “fire” in the absence of external stimuli, they become the basis for thought. Different depictions of the Cell Assembly are shown in Fig. 4.

Much has been written about the cell-assembly theory, starting with the revision by Peter Milner [133] and expanded in a number of papers [134, 135, 138]. Although Orbach [146] claimed that the idea of the cell assembly originated with Lashley, Milner [136] refutes this in no uncertain terms. There are numerous other reviews of the cell assembly that indicate its importance in theories of brain function [4, 41, 97, 122, 144, 147, 187].

### The phase sequence

A phase sequence is a temporal sequence or series of cell-assemblies which represents a chain of ideas, from sensation to thought, or a train of thought (see [84], page 92). While cell assemblies formed the basis of perceptual learning, the phase sequence was the basis of perceptual integration as envisioned by the Gestalt psychologists. The phase sequence was also the basis of associative learning (see [133], page 248). Hebb ([71], page 31) had originally used the term "Schema (“A schema of perception”) and stated that: “the schema represents some features of the process of perception”. Hebb ([71], page 39) states that “The schema implies that the difference between two perceptions derives from the mode of combination of cells, not from activity in totally different sets of cells.” The phase sequence was seen as the temporal activation of a series of cells in the schema. In his Precis, Hebb [71] had yet to articulate the cell assembly and phase sequences fully. However, as mentioned above, Hebb [73] outlined his concept of the phase sequence as a temporally organized pattern of cellular activity which could be identified with a train of thought and this paper gave a clear description of the phase sequence.

The development of these three concepts in *The Organization of Behavior* can be followed through the development of Hebb’s theory. Starting in 1932, the “Hebb synapse” was an attempt to explain Pavlov’s conditioned reflexes in terms of Sherrington’s physiology of the nervous system [58]. The cell assembly took into consideration Lashley’s [113] critiques of Pavlov by adding a mechanism for set or attention [48] and providing a mechanism derived from Lorente de Nó for neural activity to persist beyond the termination of a stimulus. The cell assembly provided the basis for perception and learning and the phase sequence provided a mechanism for linking cell assemblies to form the basis of memory, thought and imagination; for linking unrelated concepts into new ideas. As Hebb ([77], page 79) pointed out “what we are aiming at here is the solution to a psychological problem. To get psychological theory out of a difficult impasse, one must find a way of reconciling three things without recourse to animism: perceptual generalization, the stability of memory, and the instabilities of attention.”

### The autonomous central process

Hebb ([77] pages 5–10) discussed the importance of postulating neural functions that are independent of sensory stimulation. He reviews the concepts of the “central process” [91], “central motive state” [139] or “central excitatory state” [125, 172] and calls this an “autonomous central process”. Hebb ([77], page 8) proposed that the autonomous central process as defined by physiologists should be integrated with psychological theories of attitude, expectancy and set as reviewed by Sherrington [172]. Hebb cited evidence from a number of sources including Beach’s [11] paper on the neural basis of sexual behaviour, Weiss’s [188] paper on autonomous versus reflexive activity of the CNS and Jasper’s [98] paper on spontaneous electrical activity in the brain as recorded by EEG, for the knowledge of such autonomous activity in nerve cells. Hebb had known Beach and Weiss at Chicago and Jasper in Montreal and learned from all of them. Morgan ([139], page 65) pointed out that the concepts of a central excitatory state and a central inhibitory state came from Sherrington and “were conceived as the accumulation of ions at the synapse which, respectively, facilitated or inhibited synaptic transmission ([139], page 65). So it is a mystery why Hebb did not include inhibition as part of his cell assembly theory.”

Much of Hebb’s thinking about the autonomous central process (ACP) was worked out in his notes [69] in which he wrote: “ Idea of autonomous central LNCs (Lorente de Nó circuits), ACP with sensory component of organization, but presumably subject to their own physiological and anatomical determinants: normally, presumably, subject to sensory activation, but also capable of autonomous activity” (page 1). As Hebb worked out his theory, the LNC became the cell assembly and the ability of one LNC to influence another became the phase sequence. The ability of an LNC to show autonomous activity provided the basis for attention or set and for thought and imagination, the linking of two independent LNCs to create a new LNC combining both; a new thought by association of two old circuits. Using these ideas, Hebb [69] worked out a neural explanation for the Law of Effect (page 6) and the results are presented in Chapter 6 of *The Organization of Behavior* (Development of the learning capacity) which uses the concept of the cell assembly, the central autonomous process and the combination of cell assemblies into phase sequences to develop a neural explanation for associative learning.

### Inhibition

**Fig. 6:**
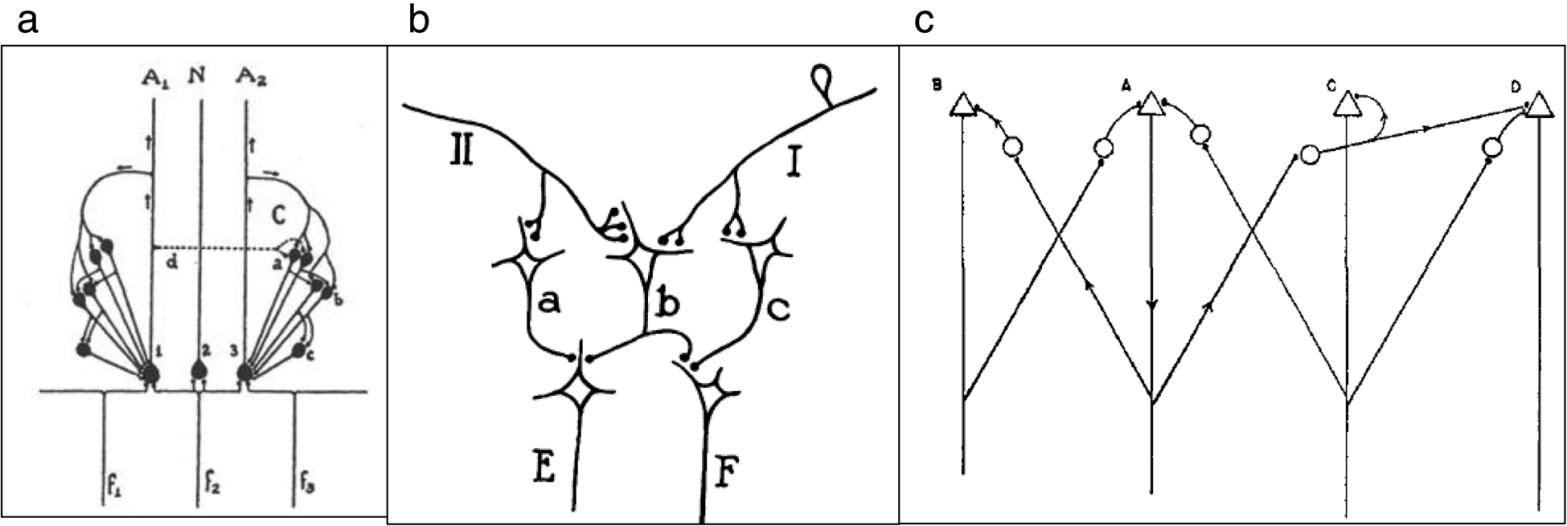
Concepts of inhibition as depicted by Lorente de Nó, Morgan and Milner. (**a**) A diagram explaining the production of reflex reversal by concurrent stimulation of two fibers (f_1_ and f_2_ or f_2_ and f_3_) from different peripheral sense organs and its maintenance by the impulses conducted by the closed chain C, after fiber f_3_, which initiated the response of cell 3, ceases conducting. Each one of the links in the closed chain represents a multiple chain of (see M in Fig. 4A of this paper). Collateral d by lowering the threshold of cell a and thus causing two impulses to cross through cell 3 in quick succession may produce inhibition, for cell 3 will acquire a high subnormal threshold. (From [124], page 231). (**b**) A diagram of neurons and synaptic connections involved in reciprocal inhibition (innervation). Excitation at the synapses is widely held to be proportional to the number of active endings. For the sake of argument, the minimal number of endings which must be active for excitation to occur is arbitrarily taken to be two. If a neuron, b, common to two pathways is switched out of one pathway when it is taken up by another, the necessary condition for reciprocal innervation would be fulfilled. Let us suppose that rhythmic stimulation of fiber I is maintaining a flexor reflex. Neurons b and c are excited, and their discharges arriving synchronously at F cause it to respond. Then let us suppose that in the course of this response an extensor reflex is set up through stimulation of fiber II. The latter can excite b in the intervals between the responses to I, because of the stronger excitation which it is able to deliver through its three endings. No discharges can result therefrom in F, as the impulses in b are out of time with those in c; and I is no longer able to excite b and c in unison, because of the raised threshold of the former. Neuron b is dominated by fiber II. Its discharges are caused to be synchronous with those in a, instead of those in c, and activity begins in E. Thus, when innervation of the extensor muscles starts it must be withdrawn from the flexor muscles. (From Gasser [46] and reprinted in Lorente de Nó [124], page 231, and Morgan [139], page 67). (**c**) Types of recurrent inhibitory connections. It is proposed that the short-axon inhibitory cells which receive recurrent collaterals from a long-axon cell have fewer inhibitory connections to that particular long-axon cell than they do to other long-axon cells in the region. When Cell A is firing it causes the inhibition of its neighbors, B, C, and D, but is not itself inhibited. In fact, because the surrounding long-axon cells cannot now be fired, there is no way in which the short-axon cells discharging onto A can be fired. Therefore, as long as A continues to fire, it protects itself from being inhibited. (From [133], page 246). **a** and **b** Reprinted from Lorente de No R. Analysis of the activity of the chains of internuncial neurons. J Neurophysiol. 1938;1:207–44 [124]. Copyright (1938), with permission from the American Physiological Society. **c** Reprinted from Milner PM. The cell assembly: mark II. Psychol Rev. 1957;64:242–52 [133]. Copyright (1957), with permission from the American Psychological Association

Although Hebb’s [58] M.A. thesis and his unpublished essay [59] stressed inhibition, there was no concept of inhibition in the cell assembly hypothesis. This has always seemed strange. In his revision of the cell assembly theory, Milner ([133], page 243) states that: “At the time when Hebb was developing his theory, many physiologists were strongly opposed to the idea of neural inhibition, largely because it was difficult to fit into the electrical theory of synaptic transmission.” But Hebb had already written a great deal about inhibition. Lorente de Nó [125] described inhibition and provided a diagram of inhibition in a closed loop. Morgan ([139], pages 66–67) described successive inhibition and reciprocal inhibition, and provided a diagram of neurons and synapses involved in reciprocal inhibition that is almost identical to that proposed by Milner [133] for the cell assembly mark II. These are shown in Fig. 6.

So, how did inhibition disappear from Hebb’s theory? In Hebb’s [69], notes he says “I am deliberately vague about when or where one LNC will (A) facilitate another, and hence make possible structural (B) facilitation; simultaneous activity not enough, because of (1) anatomic separations, (2) apparent fact that one system can inhibit or extinguish another, (3) factors of timing utilized by Lashley in his theory of repetitive circuits” (page 5). However, when Hebb [71] got into his theory of reverberating circuits in his Precis, the idea of inhibition seems to have been dropped. Whatever the reason, Milner’s [133] revision of the cell assembly theory added the concept of inhibition. In fact, Hebb [77] does consider the problem of inhibition on pages 208–215 (chapter 9). But he does not consider inhibitory synapses, even though he refers to Sherrington’s ([172], page 532) central inhibitory state (c.i.s.). For Hebb ([77], page 214), inhibition meant fatigue of excitatory pathways. He stated that “If it were safe to assume a true long-term inhibition (or equally, fatigue) in cerebral action it would greatly simplify the task of the psychologist. As matters stand, however, this is not justified.”

Someone must have convinced him that his earlier work on unconditioned and conditioned inhibition was misguided. Hebb ([84], page 101) said that “the existence of a neural inhibition was not definitely known in 1949 and was not incorporated in the theory though it would have helped. Peter Milner [133] showed how to incorporate it.” Hebb ([80] page 92) discussed neural inhibition as the prevention of a nerve from firing. According to Fairen ([42], page 436), Lorente de Nó “conceived the existence of inhibition, and wrote that internuncial neurons might be the siege of it, then he disdained this concept absolutely, and did not mention it further”. Conclusive evidence for inhibitory neurons was not available until the concept of chemical neurotransmitters was accepted and inhibitory synapses were identified [40]. From this discussion, it seems that since Lorente de Nó did not refer to inhibition, neither did Hebb.

### Chemical neurotransmitters

Hebb never mentioned chemical neurotransmitters; all of his ideas about synaptic activity were based on electrical connections between cells, although he does not explicitly state this. It is interesting that Hebb [77] cited all of the papers from the 1939 symposium on the synapse without ever mentioning the ongoing controversy between the electrical and the chemical theories of synaptic transmission that were discussed in many of these papers. The state of knowledge about synaptic transmission was summarized by Forbes ([45], page 471), who stated that “we must not think so loosely as to overlook the fact that the electrical potential which excites a tissue is the same thing whether produced by a dynamo or by a galvanic cell, and this is quite distinct from acetylcholine - a substance which can be put in a bottle, whatever its electrical properties.” However Lorente de Nó ([125], pages 420–421) was clearly opposed to the concept of chemical transmission and the reality of chemical neurotransmitters in the brain was not acknowledged until 1954 [39, 40].

Morgan ([139] pages 62–63) discussed both the humoral and electrical theories of synaptic transmission, so Hebb knew about chemical transmission, but, as noted by Morgan, the experiments to demonstrate chemical neurotransmitters in the central nervous system “have not proved conclusive” and “it is most convenient to regard transmission as electrical, for we are better acquainted with the electrical phenomena of nervous activity than the chemical, and it is easier, perhaps, to think in electrical rather than chemical terms.” ([139], page. 63). Hebb’s sister Catherine was working on the role of the neurotransmitter acetylcholine in the brain, but this work did not begin until 1951 [8, 55]. Hebb did not discuss chemical neurotransmission in *The Organization of Behavior* nor in his textbook of Psychology [80] until the fourth edition [86]. His reference to humoral mechanisms in *A textbook of Psychology* ([80], page 52) refers to estrogen.

### The relationship of instinct to learning

Hebb’s early papers from his PhD thesis were concerned with innate behaviour. For example, Hebb [62] concluded that: “In the rat, the figure-ground organization and the perception of identity in such geometrical patterns as the solid triangle, outline of triangle, and triangle circumscribed by a circle are innately determined” (page 125). Likewise, Hebb [63] referred to “an innate property of visual perception” (page 298). However, when he came to write *The Organization of Behavior*, Hebb (in his Precis of [71]) says that when he re-examined his thesis data, the dark reared rats took a mean of 129 trials while controls took only 20 trials (page 23), He has described this on page 113 of *The Organization of Behavior* and discussed how he came to make this error in his Essay on Mind ([84] page 90).

Hebb discussed the relation of instinct to learning in chapter 7 of *The Organization of Behavior*. In this chapter, Hebb considered factors that modulate learning, such as attention, motivation and emotion. He was also concerned with “the innate processes of instinct that are thought to take the place of learning” (page 140). With respect to innate or hereditary factors, Hebb ([77], pages 165–170) refers to Lashley’s [116] paper on the experimental analysis of instinct, to Beach’s [10] studies of instinct and learning in rats and to Tinbergen’s [182] paper on the objective study of innate behaviour. Hebb’s view was that it was important to distinguish innate from learned behaviour, however, he pointed out that “there is presumably no mammalian behavior that is uninfluenced either by learning or by the constitution that makes some learning easy or inevitable.” He stated that “Ultimately, our aim must be to find out how the same fundamental neural principles determine all behavior” (page 166).

Hebb [78] expanded on these ideas when he said that “there is no behaviour, beyond the level of the reflex, that is not essentially dependent on learning”, and that “no behaviour can be independent of an animal’s heredity”. Hebb’s conceptualization of innate and learned behaviour and their interaction led him to examine the factors of heredity, maturation and early learning in a series of experiments beginning with rearing rats at home in 1947. The research done by his students at McGill on environmental enrichment and impoverishment and the effects of environmental experience on perceptual and cognitive development were his attempts to understand the interaction between heredity and environment [181].

### The cell assembly versus the engram

Since the re-discovery of Richard W. Semon’s book The Mneme [167] by Schacter et al. [164], it has become popular to equate Hebb’s cell assembly with Semon’s engram [100, 185]. However Hebb did not refer to Semon in *The Organization of Behavior* and only mentioned “the engram” on page 15 of his 1945 Precis [71] (“This makes the assumption that there is a dual mechanism of the trace, a ‘dynamic’ plus a ‘structural engram.”). He used the terms “memory trace” and “mnemonic trace” (page 12) in *The Organization of Behavior*. Lashley ([118], page 3), on the other hand, made the term “engram” popular as “the locus of memory traces”. Lashley [112] did not use the term “engram” nor refer to Semon, but Lashley and Ball ([119], page 97) refer to “The engram of the maze habit” and Lashley [114] refers to maze learning ability as “the ekphore of Semon”, so he was familiar with Semon’s book. Lashley ([115], page 472) discussed the localization of the engram but dismissed Semon’s ideas by saying “Hering, Semon, Rignano and others have held that memory is a basic property of protoplasm; neurologists have been inclined to regard it as the product of some peculiar organization of nerve cells” ([115], page 457). Hebb did not refer to Semon nor the engram.

Semon’s mnemic theory was very complex and early reviews pointed out that the “mnemic principle attempted to connect memory, habit and heredity” [141]. Semon’s concept of the engram involved a number of ‘Mnemonic Principles’ applied to instincts, ontogeny, habit and ‘inherited dispositions’ and it was this latter principle of “transmission of acquired characteristics” which was “a heresy to most biologists” ([141], page 337). Today we could interpret Semon’s theory as the first attempt at an epigenetic theory of the effects of the environment on the brain and behaviour (see [30, 156, 158]). Semon’s ideas influenced many areas of biology and psychology of which I mention three: evolutionary theory, psychoanalytic theory and the search for the locus of memory in the brain.

In his presidential address to the British Association for the advancement of science, Charles Darwin’s son, Frances Darwin ([33], page 423) promoted Semon’s “mnemonic theory” of evolution over Weismann’s theory of inheritance because it “harmonizes with the facts of heredity and ontogeny”. However Darwin was critical of Semon’s theory because, although it proposed that engrams are formed in the brain by neurons, it cannot account for the formation of engrams in plants or in germ cells. Darwin [33] uses the terms ‘nerve-engrams’ and ‘germinal engrams’, and is concerned with the mechanisms through which engrams could be inherited and develop to control instinctive behaviour. He says “If we are to believe that an individual habit may be inherited and appear as an instinct, the repetition of the habit will not merely mean changes in the central nervous system, but also corresponding changes in the germ cells” and alludes to the epigentic nature of Semon’s theory ([33], page 423). Dendy ([35], page 371) examined Semon’s “mnemic theory of heredity” and noted that it “endeavours to explain the phenomenon of inheritance as due to a kind of unconscious memory”. He goes on to say that Semon’s “mnemonic theory, which is based upon the inheritance of acquired characters, naturally does not appeal to those who deny the possibility of such inheritance.”

Brock ([17], page 106) was one of the earliest to associate Semon’s ideas with psychiatry and was interested in “the existence in the cell of some sort of ‘unconscious memory’ conveying a tradition of ancestral behaviour”. In a footnote, Brock ([17], page 106) states that “Semon has devised an imposing terminology of Greek origin, and his work has been elaborated with characteristic German thoroughness. Although he is anxious to rebut any charge of ‘vitalism’ and insists that his ‘engram’ is essentially something ‘material’.” Jelliffe [99] proposed the use of Semon’s ideas in psychoanalytic theory and focused on Semon’s concept of energy and how, even though Semon criticized Freud, their ideas might have been compatible if Semon had lived longer. According to Jelleffe ([99], page 333) “Semon’s theory of the “mneme” is that all the organic phenomena of reproduction, repetition of bodily form or of dynamic experiences, are expressions of one and the same energic process. These mnemic phenomena are something more than the repetition to be observed in the organic world. They represent a reproduction or repetition in which the new conditions are never quite the same as the original ones. This is expressed in the term engram.” Jelleffe [99] ends with the comment that although Semon did not believe in Freud’s idea of “a store of infantile memories”, that “Semon and Freud might have found themselves closer in that illuminating union of thought which would make even clearer the correlations of energic processes with the subjective experiences of consciousness.” (page 341). Mott [141] described Semon’s Three Primal Instincts: the Instinct of Self preservation, the Sex Instinct, and the Gregarious or Social Instinct, all of which would appeal to Freudians.

Gordon [49] used Semon’s concept of the engram to try to understand the neural basis of nervous disorders and Davies [34] referred to “Semon’s engram” when discussing the neural basis of hallucinations. In discussing memory and heredity, Reiser ([157], page 704) stated that “Semon’s concept of phyletic memory, as exhibited by instincts, habits, and ‘pattern reaction’, is based on the idea that etchings in the protoplasm by external stimuli leave certain reaction tendencies called engrams (engrammes)”. Finally in the literature on behaviourism, Semon’s mnemic theory provided a neural basis for the formation of associations. Although Mursell ([143], page 14) was critical of Semon’s concept of the engram, he argued that terms such as ‘engram’ or ‘neurogram’ (a neural engram) were important because “in mnemic causation we have the principle of integration that is required by objective psychology”.

However, when referring to memory in the brain, the concept of the engram gradually became separated from Semon’s overall mneme theory. For example, Sherrington ([171], page 349) referred to “the oft-remarked trend ... for a reaction to leave behind itself a trace, an engram, a memory, the reflex engram, and the mental memory”, without mentioning Semon. Campion [28] referred to “the Semon-engram” and Black [14] to “engram complexes” without referring to Semon at all. Rigano ([161], page 121) referred to “an engram (to use Semon’s terminology)” and Muenzinger [142] referred to the engram “as studied by Lashley” and did not mention Semon. Likewise Tolman ([183], p. 31) refers to Lashley when he discusses “the mnemonic process or engram” as the neural localization of memory.

Thus, Semon’s concept of the engram was enmeshed in a complex theory of heredity, instinct, learning and evolution, and behavioural psychologists who studied the neural basis of memory slowly dissociated the use of the term “engram” from Semon’s overall theory and used it to mean the neural location of a memory. Holt ([92], page 30) noted that “The evidence of engrams, inherited instinct, or mneme (R. Semon) is singularly scanty”. However, he then went on to use the terms ‘neural engram’ and ‘neurogram’ without referring to Semon and Hebb made reference to Hoyt in his PhD thesis and early papers [62, 63]. If one examines Holt’s [92] book, there is the seed of Hebb’s synapse theory. Holt ([92], page 34) stated that “Engrams or special pathways for nerve impulses, are inscribed in this diffuse network [of neurons] by a lowering of the resistance of some, as compared with other, synaptic junctions. Though the electro-chemistry of this process is imperfectly understood, the fact is sufficiently attested that every passage of a nervous impulse across the junctional tissue between two neurones (the synapse) lowers the resistance of that tissue to the passage of all subsequent nervous impulses.” In a footnote, Holt ([92], page 34) noted that Lashley [111] “has raised objections to the view that synaptic resistance is lowered by the passage of nervous impulses”.

Hebb was, therefore, aware of the term ‘engram’ from working with Lashley and had read Holt [92], but since Hebb believed that his research showed innate visual abilities, he dismissed Holt’s argument that there was no innate organization of sensory or motor process [62, 63]. It is clear that Hebb reversed his position as he was writing his notes for *The Organization of Behavior* and came to believe that learning was essential for the development of perception. One might hypothesize that he was put off the ‘engram’ concept and put on to the concept of synaptic change by reading Holt [92], but he does not refer to Holt in *The Organization of Behavior*. He replaced the theory of the engram with his cell assembly theory.

Takamiya et al. [177] discuss the similarities and differences between the cell assembly and engram. They point out that the engram was seen as a fixed location for a memory over one or more neurons, while the cell assembly provides a mechanism for the formation of the memory trace and a mechanism for the modification of memory traces. Takamiya et al. [177] have equated Hebb cell assemblies with the concept of the ‘engram’ and say that “Populations of engram cells could be considered as cell assemblies encoding memory engrams.” They say that an ‘engram’ is “a collection of neurons that is activated to recall a memory” but “engram cells are not thought of as a unit necessary to form an engram”. They say that the “synchronous firing of neurons to encode a memory consists of a cell assembly being defined as functionally connected neurons via synchronous firing.” Thus, the cell assembly creates the engram. And so the story has come full circle: Hebb’s concept of synaptic change forms a cell assembly, which creates a long-term memory [1] and this constitutes the “engram”.

## The importance of the Organization of Behavior today

There are few areas of psychology and neuroscience today that are not influenced by Hebb’s ideas of synaptic plasticity and cell assemblies [21, 154]. The synaptic theory of learning and memory developed by Hebb remains a cornerstone of modern neuropsychological thought [109, 153, 169, pages 162–166]. Hebb’s ideas are used in research on the neural basis of cognition [122, 155]; machine intelligence [53], robotic engineering and artificial intelligence [36, 43, 152, 180]. Here I will discuss the importance of Hebb’s ideas for understanding the role of synaptic change and the development of cell assemblies and phase sequences in the neurophysiology of learning and memory and in neurocomputing.

### The neurophysiology of learning and memory

The proposal that long-term potentiation (LTP) was a synaptic model of memory [15] led to a number of examinations of Hebb’s concept of synaptic plasticity [32, 166, 174, 176]. The concept of spike timing dependent plasticity (STDP) then built on the concept of the Hebb synapse, resulting in the term “Hebbian STDP” [25]. The Dynamic Hebbian Learning Model (dynHebb) has been developed to account for the complexities of STDP [145]. McNaughton [131] wrote about how Hebb’s theory stimulated his research on LTP and memory and Andersen et al. ([5], page 188) stated that “Hebbian plasticity, as represented by long-term potentiation (LTP) and long-term depression (LTD) of synapses, has been the most influential hypothesis to account for encoding of memories.”

Hebb’s cell assembly theory has stimulated research on neural networks [122, 147, 148] and the molecular mechanisms underlying the cell assembly [97, 155, 187]. Harris ([52], page 264) stated that “One of the most influential theories for cortical function is the ‘cell assembly hypothesis’ first proposed over half a century ago [77]” and Harris [51] proposed four experimental tests for the temporal organization of cell assemblies. Eichenbaum [41] proposed that cell assemblies be studied as “units of information processing” to guide research on “the structure and organization of neural representations in perception and cognition”.

**Fig. 7:**
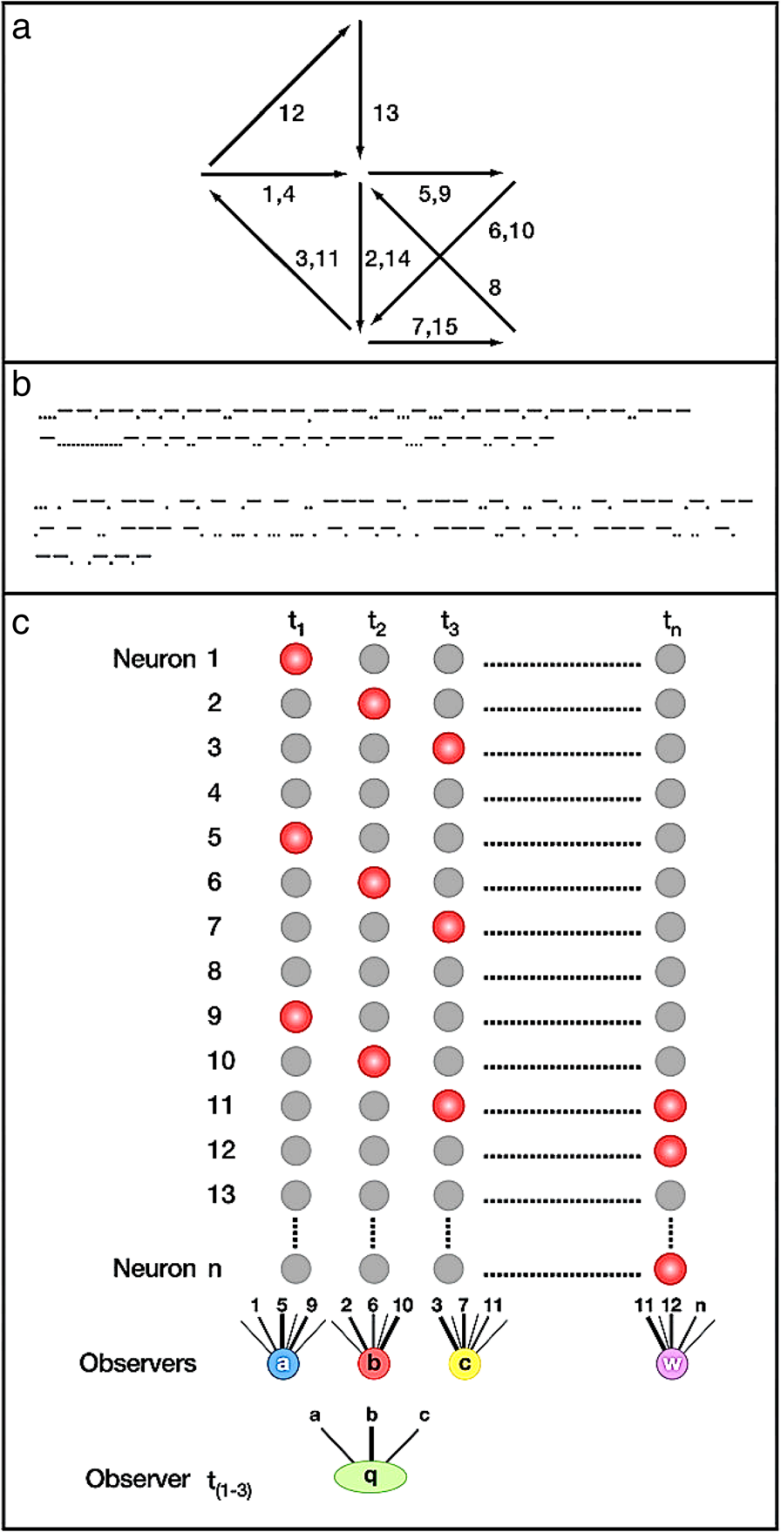
Buzsaki’s [26] extension of Hebb’s Cell Assembly and Phase Sequence. (**a**) Hebb’s [77] diagram of a cell assembly. Arrows represent transitions between individual assemblies. The direction of activity flow across assemblies (edges) is determined by the stronger synaptic strengths among assembly members relative to other connections (not shown). The same assembly can participate in a sequence more than once (e.g., pathway 1, 4 indicates recurring transitions). (**b**) Top: long sequence of two characters (e.g., dot and dash). Its embedded information is virtually impossible to recover. Bottom: same exact sequence as above after adding syntactic segmentation (space = stop-start punctuation) between the short strings of characters. The Morse code message reads: “segmentation of information is essence of coding.” By analogy, segmentation or “chunking” of neuronal assemblies can be brought about by salient external stimulus sequences, brain-initiated, modality-specific synchronizing- blanking mechanisms, internally generated oscillations, or other syntactical mechanisms. (**c**) Reader-defined cell assemblies. Neurons that fire within the time integrating window of a reader mechanism define an assembly (irrespective of whether assembly members are connected synaptically or not). Readers a, b, c, and w may receive inputs from many neurons (1 to n) by way of synapses differing in strength but respond only to a combination of spiking neurons to which they are most strongly connected (e.g., reader a responds preferentially to co-firing of neurons 1, 5, and 9 at t1, even though it may be synaptically innervated by neurons 2, 6, and 10 as well; at t2, neuron b fires in response to the discharge of neurons 2, 6, and 10). Synaptic strengths between neurons vary as a function of the spiking history of both postsynaptic and presynaptic neuron (short-term plasticity). The response of the reader neuron, therefore, depends on both the identity of the spiking upstream neurons and the constellation of current synaptic weights (“synapsembles”). Reader mechanism q has a longer time integrator and, therefore, can link together assemblies to neural “words,” reading out a new quality not present in the individual representations of a, b, and c. Reprinted from Buzsaki G. Neural syntax: cell assemblies, synapsembles, and readers. Neuron. 2010;68:362–85 [26] Copyright (2010), with permission from Elsevier

Buzsaki [26] defined the cell assembly as the neural syntax of the brain and suggested ways in which the neural organization of cell assemblies could be understood in the context of both brain function and brain-machine interfaces. He proposed that cell assemblies were linked by “dynamically changing constellations of synaptic weights” which he called “synapsembles” and suggested that the objective identification of the cell assembly requires a temporal framework and a reader mechanism which can integrate the activity of cell assemblies over time (See Fig. 7). The result of such thinking has led to the consideration of Hebbian cell assemblies as the basis for “semantic circuits” which define “the cortical locus of semantic knowledge” and to the development of neurocomputational models of brain function [184].

**Fig. 8:**
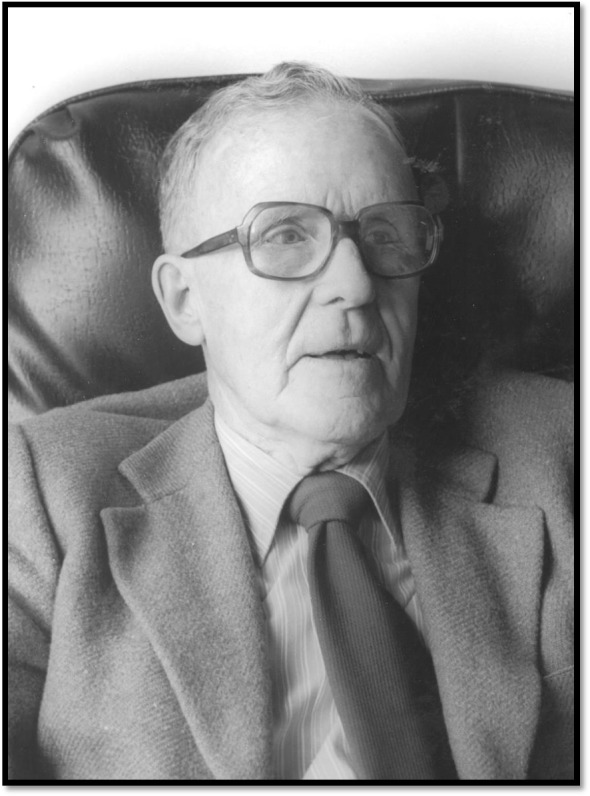
Donald O. Hebb in 1979. Photo taken by Richard Brown. It was used as the frontspiece in the republication of *The Organization of Behavior* in 2002 by Erlbaum. Reprinted with permission from Richard E. Brown

Finally, Hebb’s concept of phase sequences as synchronized sets of cell assemblies has been examined by recording action potentials from the hippocampus and cortex of actively behaving rats [4]. The results suggest that the cell assemblies are the building blocks of neural representations, while the phase sequences that link cell assemblies are modifiable by new experiences, modulating the neural connections of cognition and behaviour. This approach has been used to apply Hebbian learning and cell assemblies to the construction of neurocomputational models of language learning which simulate the brain mechanisms of word meaning in “semantic hubs” [184].

### Neurocomputing

The concept of Hebbian learning is used in neurocomputing and the development of artificial neural networks [108]. The mathematical definition of the change in activity at a Hebb synapse through “synaptic scaling” has allowed for the quantitative definition of a Hebbian Cell Assembly [179] for use in robotics and artificial intelligence. Virtual Cell Assembly Robots (CABots), have been built using cell assemblies as the basis of short and long term artificial memories [27, 96] and the cell assembly has been proposed as the basis for computer simulation of human brain function [95].

Finally, the existence of this meeting on The Hebb synapse 70 years later, and the papers in this special issue point to the lasting importance of Hebb’s ideas (Fig. 8). The papers at this meeting discuss long-term and short-term potentiation, the plasticity of individual synapses, neuromodulation of Hebbian synapses, the importance of pre-and post-synaptic plasticity in learning, and the role of the synapse in developing an engram. To mention a few: Graham Collingridge’s paper on Hebb synapses and beyond; Ole Paulson’s paper on Neuromodulation of Hebbian synapses and Zahid Padamsey’s presentation on a new framework for Hebbian plasticity in the hippocampus. Given the importance of Hebb’s ideas for the understanding cognitive processes [41, 177], and the focus of this meeting on synaptic mechanisms in learning and memory, it is no surprise that Hebb is a hero of the engram [101].

## Data Availability

Data sharing not applicable to this article as no datasets were generated or analysed during the current study. All of Hebb's unpublished papers and letters are available from McGill University Archives, Montreal, Quebec, file MG1045.

## Abbreviations

ACA: Autonomous central action
ACP: Autonomous central process
C: Closed
CABots: Cell assembly robots
CS: Conditioned stimulus
K: Köhler
KSL: Karl Spencer Lashley
L: Lashley
LNC: Lorente de Nó circuit
LTD: Long-term depression
LTP: Long-term potentiation
M: Multiple
M.b.: Midbrain
Med: Medulla
MNI: Montreal Neurological Institute
MS: Manuscript
O of B: Organization of Behaviour
Oc.n.: Oculomotor nuclei
P.: Pons
Pp: Paragraphs
Rcs: Conditioned response
Rev.: Reverberatory
R_uncS_: Unconditioned response
STDP: Spike timing dependent plasticity
U_ncS_: Unconditioned stimulus
V: Vestibular nerve

## Footnotes

1 The other four books were *The concept of mind* by Gilbert Ryle, *Perception and communication* by Donald Broadbent, *Evolution of the brain and intelligence* by Harry Jerison and *Images of mind* by Michael Posner and Marcus Raichle

## References

[1] Abraham WC, Jones OD, Glanzman DL (2019). Is plasticity of synapses the mechanism of long-term memory storage?. NPJ Sci Learn.

[2] Adams P (1998). Hebb and Darwin. J Theor Biol.

[3] Allport FH (1955). Theories of perception and the concept of structure.

[4] Almeida-Filho DG, Lopes-dos-Santos V, Vasconcelos NAP, Miranda JGV, Tort ABL, Ribeiro S (2014). An investigation of Hebbian phase sequences as assembly graphs. Front Neural Circuits.

[5] Andersen N, Krauth NK, Nabavi S (2017). Hebbian plasticity *in vivo*: relevance and induction. Curr Opin Neurobiol.

[6] Attneave F (1950). The organization of behavior: a neuropsychological theory. Amer J Psych.

[7] Babichev A, Cheng S, Dabaghian YA (2016). Topological schemas of cognitive maps and spatial learning. Front Comput Neurosci.

[8] Balfour WE, Hebb CO (1951). Effects of choline, acetate and citrate on the activity of the choline acetylating system of mammalian brain. Nature.

[9] Bartlett FC. Remembering: a study in experimental and social psychology. London: Cambridge University Press; 1932.

[10] Beach FA (1939). The neural basis of innate behaviour. III Comparison of learning ability and instinctive behavior in the rat. J Comp Psychol.

[11] Beach FA (1942). Analysis of factors involved in the arousal, maintenance and manifestation of sexual excitement in male animals. Psychosom Med.

[12] Berlucchi G, Buchtel HA (2009). Neuronal plasticity: historical roots and evolution of meaning. Exp Brain Res.

[13] Bijoch L, Borczyk M, Czajkowski R. Bases of Jerzy Konorski’s theory of synaptic plasticity. Eur J Neurosci. 2019. 10.1111/ejn.14532. 10.1111/ejn.14532 31368131

[14] Black C (1928). Note on the nature of intelligence. Br J Psychol.

[15] Bliss TV, Collingridge GL (1993). A synaptic model of memory: long-term potentiation in the hippocampus. Nature.

[16] Boring EG. Letter to Hebb dated 25 July 1946: Held in the Hebb archives at McGill University, Montreal, Quebec, file MG1045.

[17] Brock AJ (1913). The psychological conception of disease. Edinb Med J.

[18] Brown RE (2006). The life and work of Donald Olding Hebb. Acta Neurol Taiwanica.

[19] Brown RE (2007). The life and work of Donald Olding Hebb: Canada's greatest psychologist. Proc Nova Scotian Inst Sci.

[20] Brown RE (2016). Hebb and Cattell: the genesis of the theory of fluid and crystallized intelligence. Front Hum Neurosci.

[21] Brown RE, Kolb B, Whishaw I (2017). Revisiting Hebb: The Organization of Behavior. Chapter 7. Brain and behaviour: revisiting the classic studies.

[22] Brown RE, Milner PM (2002). Foreword to the Erlbaum edition, D.O.Hebb, The Organization of Behavior.

[23] Brown RE, Milner PM (2003). The legacy of Donald O. Hebb: more than the Hebb synapse. Nat Rev Neurosci.

[24] Bruce D (1996). Lashley, Hebb, connections, and criticisms. Can Psychol.

[25] Brzosko Z, Mierau SB, Paulsen O (2019). Neuromodulation of spike-timing-dependent plasticity: past, present, and future. Neuron.

[26] Buzsaki G (2010). Neural syntax: cell assemblies, synapsembles, and readers. Neuron.

[27] Byrne E, Huyck C (2010). Processing with cell assemblies. Neurocomputing.

[28] Campion GG (1925). The neural sub-strata of reflective thought. An outlined integration of the psychological and neural elements. Brit J Med Psychol.

[29] Cattell RB (1943). The measurement of adult intelligence. Psych Bulletin.

[30] Cavalli G, Heard E (2019). Advances in epigenetics link genetics to the environment and disease. Nature.

[31] Centonze D, Siracusano A, Calabresi P, Bernardi G (2004). The project for a scientific Psychology (1895): a Freudian anticipation of LTP-memory connection theory. Brain Res Brain Res Rev.

[32] Cooper SJ (2005). Donald O. Hebb’s synapse and learning rule: a history and commentary. Neurosci Biobehav Rev.

[33] Darwin F (1908). Inaugural address. Nature.

[34] Davies AE (1926). An interpretation of mental symptoms of dementia praecox. J Abnorm Psychol.

[35] Dendy A (1912). The mnemic theory of heredity. Nature.

[36] Der R. In search for the neural mechanisms of individual development: behavior-driven differential Hebbian learning. Front Robot AI. 2016. 10.3389/frobt.2015.00037.

[37] Dewsbury DA (2002). The Chicago five: a family group of integrative psychobiologists. Hist Psychol.

[38] Dewsbury DA (2006). Monkey farm: a history of the Yerkes Laboratories of Primate Biology, Orange Park, Florida 1930–1965.

[39] Eccles JC (1976). From electrical to chemical transmission in the central nervous system. Notes Rec R Soc London.

[40] Eccles JC (1990). Developing concepts of the synapses. J Neurosci.

[41] Eichenbaum H (2018). Barlow versus Hebb: when is it time to abandon the notion of feature detectors and adopt the cell assembly as the unit of cognition?. Neurosci Lett.

[42] Fairén A (2007). Cajal and Lorente de Nó on cortical interneurons: Coincidences and progress. Brain Res Rev.

[43] Feldotto B, Walter F, Röhrbein F, Knoll A (2018). Hebbian learning for online prediction, neural recall and classical conditioning of anthropomimetic robot arm motions. Bioinspir Biomim.

[44] Flyn C (2017). The best books on cognitive neuroscience: recommended by Dick Passingham.

[45] Forbes A (1939). Problems of synaptic function. J Neurophysiol.

[46] Gasser HS (1937). The control of excitation in the nervous system. Bull N Y Acad Med.

[47] Ghosh VE, Gilboa A (2014). What is a memory schema? A historical perspective on current neuroscience literature. Neuropsychologia.

[48] Gibson JJ (1941). A critical review of the concept of set in contemporary experimental psychology. Psychol Bull.

[49] Gordon RG (1923). The nervous child. J Neurol Psychopathol.

[50] Haider B (2008). Contributions of Yale neuroscience to Donlad O. Hebb's *Organization of Behavior*. Yale J Biol Med.

[51] Harris KD (2005). Neural signatures of cell assembly organization. Nat Rev Neurosci.

[52] Harris KD (2012). Cell assemblies of the superficial cortex. Neuron.

[53] Hawkins J (2004). On Intelligence.

[54] Head H, Holmes G (1911). Sensory disturbances from cerebral lesions. Brain.

[55] Hebb CO (1957). Biochemical evidence for the neural function of acetylcholine. Physiol Rev.

[56] Hebb CO (1972). Biosynthesis of acetylcholine in nervous tissue. Physiol Rev.

[57] Hebb DO (1930). Elementary school methods. Teach Mag.

[58] Hebb, D.O. 1932. Conditioned and unconditioned reflexes and inhibition. Available from McGill University Archives, Montreal, Quebec, file MG1045.

[59] Hebb, D. O. 1934. The interpretation of experimental data on neural action. Available from McGill University Archives, Montreal, Quebec, file MG1045.

[60] Hebb, D. O. 1934. Scientific methods in psychology: a theory of epistemology based on objective methods in psychology. Unpublished manuscript.

[61] Hebb DO. The innate organization of visual perception in the rat: PhD Thesis, Harvard University, Cambridge, Mass; 1936. March 1936. [Available on microfilm from Harvard University Archives].

[62] Hebb DO (1937). The innate organization of visual activity: I. Perception of figures by rats reared in total darkness. J Genet Psychol.

[63] Hebb DO (1937). The innate organization of visual activity. II Transfer of response in the discrimination of brightness and size by rats reared in total darkness. J Comp Psychol.

[64] Hebb DO (1938). Studies of the organization of behavior. I. Behavior of the rat in a field orientation. J Comp Psychol.

[65] Hebb DO (1938). Studies of the organization of behavior. II. Changes in the field orientation of the rat after cortical destruction. J Comp Psychol.

[66] Hebb DO (1939). Intelligence in man after large removals of cerebral tissue: report of four left frontal lobe cases. J Gen Psychol.

[67] Hebb DO (1939). Intelligence in man after large removals of cerebral tissue: defects following right temporal lobotomy. J Gen Psychol.

[68] Hebb DO (1942). The effects of early and late brain injury upon test scores, and the nature of normal adult intelligence. Proc Am Phil Soc.

[69] Hebb, D. O. 1944. 4 concept speculations. Unpublished typed notes, 5 pages dated "about June '44". McGill University archives, MG 1045.

[70] Hebb, D. O. 1945. Structure and origin of concept speculation. Four pages of hand written notes dated "written spring '45". McGill University archives, MG 1045.

[71] Hebb, D. O. 1945. Precis: the structure of a set of neuropsychological speculations. 25 pages of typed notes, dated "March-July '45". McGill University archives, MG 1045.

[72] Hebb, D. O. 1945. A schema of perception. 23 pages of typed notes (numbered pages 27-49), no date, but they appear to be part of the Precis [71]. Available from McGill University Archives, Montreal, Quebec, file MG1045.

[73] Hebb DO (1946). On the nature of fear. Psychol Rev.

[74] Hebb, D. O. 1946. Carbon of most of the original MS of my book *The Organization of Behavior* (while the term "lattice" was still used instead of "cell assembly"); with 2 pages of Lashley's criticisms, at the front. Unpublished manuscript 251 pages. McGill University archives MG 1045.

[75] Hebb DO (1947). Spontaneous neurosis in chimpanzees; theoretical relations with clinical and experimental phenomena. Psychosom Med.

[76] Hebb DO (1947). The effects of early experience on problem solving at maturity. Am Psychol.

[77] Hebb DO (1949). The organization of behavior; a neuropsychological theory.

[78] Hebb DO (1953). Heredity and environment in mammalian behaviour. Br J Anim Behav.

[79] Hebb DO (1955). Drives and the C.N.S. (conceptual nervous system). Psychol Rev.

[80] Hebb DO (1958). A textbook of Psychology.

[81] Hebb DO, Koch S (1959). A neuropsychological theory. Psychology: A study of a science (Vol. 1).

[82] Hebb DO (1976). Physiological learning theory. J Abnorm Child Psychol.

[83] Hebb DO, Lindzey G (1980). D. O. Hebb. A history of psychology in autobiography. Vol. VII.

[84] Hebb DO (1980). Essay on mind.

[85] Hebb, D.O. 1981. This is how it was: fifty years in psychology, forty years a psychologist. Presentation at the Canadian Psychology association meeting, Available from McGill University Archives, Montreal, Quebec, file MG1045.

[86] Hebb DO, Donderi DC (1987). Textbook of Psychology.

[87] Hebb DO, Morton NW (1944). Note on the measurement of adult intelligence. J Gen Psychol.

[88] Hebb DO, Penfield W (1940). Human behavior after extensive bilateral removal from the frontal lobes. Arch Neurol Psychiatr.

[89] Hebb DO, Williams KA (1946). Method of rating animal intelligence. J Gen Psychol.

[90] Hilgard ER (1956). Theories of learning.

[91] Hilgard ER, Marquis DG (1940). Conditioning and learning.

[92] Holt EB (1931). Animal drive and the learning process, an essay toward radical empiricism.

[93] Hull CL (1943). Principles on behavior: an introduction to behavior theory.

[94] Humphrey G (1940). The problem of the direction of thought. British Journal of Psychology. Gen Sect.

[95] Huyck CR (2020). A neural cognitive architecture. Cogn Syst Res.

[96] Huyck C, Mitchell I (2018). CABots and other neural agents. Front Neurorobot.

[97] Huyck CR, Passmore PJ (2013). A review of cell assemblies. Biol Cybern.

[98] Jasper HH (1937). Electrical signs of cortical activity. Psychol Bull.

[99] Jelliffe SE (1923). The Mneme, the engram and the unconscious. Richard Semon: his life and work. J Nerv Ment Dis.

[100] Josselyn SA, Köhler S, Frankland PW (2015). Finding the engram. Nat Rev Neurosci.

[101] Josselyn SA, Köhler S, Frankland PW (2017). Heroes of the engram. J Neurosci.

[102] Koch S (1959). Psychology: a study of a science, 7 volumes.

[103] Koffka, K. Principles of Gestalt Psychology. New York: Harcourt Brace; 1935.

[104] Köhler W (1929). Gestalt Psychology.

[105] Kolb B (2003). The impact of the Hebbian learning rule on research in behavioural neuroscience. Can Psychol.

[106] Konorski J (1948). Conditioned reflexes and neuron organization.

[107] Kuhn MH (1950). Hebb, D. O. organization of behavior: a neuropsychological theory. Ann Amer Acad Pol Soc Sci.

[108] Kuriscak E, Marsalek P, Stroffek J, Toth PG (2015). Biological context of Hebb learning in artificial neural networks, a review. Neurocomputing.

[109] Langille JJ, Brown RE (2018). The synaptic theory of memory: a historical survey and reconciliation of recent opposition. Front Syst Neurosci.

[110] Lansner A (2009). Associative memory models: from the cell-assembly theory to biophysically detailed cortex simulations. Trends Neurosci.

[111] Lashley KS (1924). Studies of cerebral function in learning VI. The theory that synaptic resistance is reduced by the passage of the nerve impulse. Psychol Rev.

[112] Lashley KS (1929). Brain mechanisms and intelligence: a quantitative study of injuries to the brain.

[113] Lashley KS (1930). Basic neural mechanisms in behavior. Psychol Rev.

[114] Lashley KS (1933). Integrative functions of the cerebral cortex. Physiol Rev.

[115] Lashley KS, Murchison C (1934). Chapter 10. Learning III. Nervous mechanisms in learning. A Handbook of General Experimental Psychology.

[116] Lashley KS (1938). Experimental analysis of instinctive behavior. Psychol Rev.

[117] Lashley KS (1949). Persistent problems in the evolution of mind. Q Rev Biol.

[118] Lashley KS (1950). In search of the engram. Society for Experimental Biology symposium No. 4: physiological mechanisms in animal behaviour.

[119] Lashley KS, Ball J (1929). Spinal conduction and kinesthetic sensitivity in the maze habit. J Comp Psychol.

[120] Lashley KS, Russell JT (1934). The mechanism of vision. XI A preliminary test of innate organization. J Gen Psychol.

[121] Leeper R (1950). The organization of behavior: a neuropsychological theory (review). J Abnorm Soc Psychol.

[122] Li M, Liu J, Tsien JZ (2016). Theory of connectivity: nature and nurture of cell assemblies and cognitive computation. Front Neural Circuits.

[123] Lorente de No R (1938). Synaptic stimulation of motoneurons as a local process. J Neurophysiol.

[124] Lorente de No R (1938). Analysis of the activity of the chains of internuncial neurons. J Neurophysiol.

[125] Lorente de No R (1939). Transmission of impulses through cranial motor nuclei. J Neurophysiol.

[126] Lorente de No R, Fulton JF (1943). Cerebral cortex: architecture. Physiology of the nervous system.

[127] Maier NRF, Schneirla TC (1935). Principles of animal Psychology.

[128] Martinez LM, Gil FT (2003). Contributions to the history of Psychology CXIX: the Spanish neurohistological school's legacy: Cajal and Lorente de No. Psychol Rep.

[129] Maurer AP, Burke SN, Lipa P, Skaggs WE, Barnes CA (2012). Greater running speeds result in altered hippocampal phase sequence dynamics. Hippocampus.

[130] McBride AF, Hebb DO (1948). Behavior of the captive bottle-nose dolphin, *Tursiops truncatus*. J Comp Physiol Psychol.

[131] McNaughton BL (2003). Long-term potentiation, cooperativity and Hebb's cell assemblies: a personal history. Phil trans R Soc Lond B.

[132] Milner B, Squire LR (1998). Brenda Milner. The history of neuroscience in autobiography.

[133] Milner PM (1957). The cell assembly: mark II. Psychol Rev.

[134] Milner PM (1993). The mind and Donald O. Hebb. Sci Am.

[135] Milner PM (1999). The autonomous brain.

[136] Milner, P. M. 1999. Cell assemblies: Whose idea? Psycoloquay: 10, #53 LashleyHebb (4).

[137] Milner PM (2003). A brief history of the Hebbian learning rule. Can Psychol.

[138] Milner PM (2006). Trains of neural thought. Can Psychol.

[139] Morgan CT (1943). Physiological Psychology.

[140] Moss, F.A. 1942. Comparative Psychology, Revised edition. New York: Prentice Hall. This book has chapters by Edward L Thorndike, R.H. Waters, Calvin P. Stone, F.A. Moss, Paul E. Fields, Donals G Marquis, Howard S. Liddell, W. T. Heron, Kenneth W. Spence, Robert C Tryon and Otto L. Tinklepaugh.

[141] Mott F (1923). The mnemic principle applied to biology and psychology. Br Med J.

[142] Muenzinger KF (1928). Plasticity and mechanization of the problem box habit in Guinea pigs. J Comp Psychol.

[143] Mursell J (1924). The principle of integration in objective Psychology. Am J Psychol.

[144] Nadel L, Maurer AP. Recalling Lashley and reconsolidating Hebb. Hippocampus 2018 Sep 14. 2018. 10.1002/hipo.23027. 10.1002/hipo.23027 PMC6417981 30216593

[145] Olde Scheper TV, Meredith RM, Mansvelder HD, van Pelt J, van Ooyen A. Dynamic Hebbian cross-correlation learning resolves the spike timing dependent plasticity conundrum. Front Comput Neurosci. 2018;11:119. 10.3389/fncom.2017.00119. 10.3389/fncom.2017.00119 PMC5768644 29375358

[146] Orbach J (1998). The neuropsychological theories of Lashley and Hebb.

[147] Palm G (1981). Towards a theory of cell assemblies. Biol Cybernetics.

[148] Palm G, Knoblauch A, Hause F, Schüz A (2014). Cell assemblies in the cerebral cortex. Biol Cybern.

[149] Pavlov IP (1927). Conditioned Reflexes.

[150] Pavlov IP, Koshtoyants KS (1903). The problem of the study of higher nervous activity and the ways of its experimental solution. Published in English in I.P. Pavlov: Selected Works.

[151] Pavlov IP (1932). The reply of a physiologist to psychologists. Psych Rev.

[152] Parisi GI, Kemker R, Part JL, Kanan C, Wermter S (2019). Continual lifelong learning with neural networks: a review. Neural Netw.

[153] Poo M, Pignatelli M, Ryan TJ, Tonegawa S, Bonhoeffer T, Martin KC, Rudenko A, Tsai L-H, Tsien RW, Fishell G, Mullins C, Gonçalves JT, Shtrahman M, Johnston ST, Gage FH, Dan Y, Long J, Buzsáki G, Stevens C (2016). What is memory? The present state of the engram. BMC Biol.

[154] Posner MI, Rothbart MK (2004). Hebb’s neural networks support the intergration of psychological science. Can Psychol.

[155] Pulvermüller F, Garagnani M, Wennekers T (2014). Thinking in circuits: toward neurobiological explanation in cognitive neuroscience. Biol Cybern.

[156] Qureshi IA, Mehler MF (2018). Epigenetic mechanisms underlying nervous system diseases. Handb Clin Neurol.

[157] Reiser OL (1931). Biological relativity 1. Psychic regression and biology. J Philos.

[158] Rensing L, Koch M, Becker A (2009). A comparative approach to the principal mechanisms of different memory systems. Naturwissenschaften.

[159] Riesen AH (1947). The development of visual perception in man and chimpanzee. Science.

[160] Riesen AH, von Senden M (1959). Appendix (1959). Significance of the work for related disciplines. (i) Psychology. Space and sight: the perception of space and shape in the congenitally blind before and after operation.

[161] Rignano E (1928). The psychological theory of form. Psychol Rev.

[162] Rosenzweig MR (1998). Some historical background of topics in this conference. Neurobiol Learn Mem.

[163] Sakurai Y, Osako Y, Tanisumi Y, Ishihara E, Hirokawa J, Manabe H (2018). Multiple approaches to the investigation of cell assembly in memory research—present and future. Front Syst Neurosci.

[164] Schacter DL, Eich JE, Endel T (1978). Richard Semon's theory of memory. J Verbal Learn Verbal Behav.

[165] Schott GD (2011). Freud's project and its diagram: anticipating the Hebbian synapse. J Neurol Neurosurg Psychiatry.

[166] Sejnowski TJ (2003). The once and future Hebb synapse. Can Psychol.

[167] Semon RW (1923). The mneme.

[168] Seung HS (2000). Half a century of Hebb. Nat Neurosci Suppl.

[169] Shepherd GM (2010). Creating modern neuroscience: the revolutionary 1950's.

[170] Sherrington CS (1906). Integrative action of the nervous system.

[171] Sherrington C (1922). Some aspects of animal mechanism. Nature..

[172] Sherrington CS (1925). Remarks on some aspects of reflex inhibition. Proceedings of the Royal Society of London.

[173] Skinner BF (1938). The behavior of organisms: an experimental analysis.

[174] Spatz HC (1996). Hebb’s concept of synaptic plasticity and neuronal cell assemblies. Behav Brain Res.

[175] Spence KW (1937). The differential response in animals to stimuli varying within a single dimension. Psychol Rev.

[176] Sweatt JD (2016). Neural plasticity and behavior -- sixty years of conceptual advances. J Neurochem.

[177] Takamiya S, Yuki S, Hirokawa J, Manabe H, Sakurai Y. Dynamics of memory systems. Neurosci Res. 2019, 2019. 10.1016/j.neures.2019.03.005. 10.1016/j.neures.2019.03.005 30940458

[178] Telford CW (1931). The refractory phase of voluntary and associative responses. J Exp Psychol.

[179] Tetzlaff C, Dasgupta S, Kulvicius T, Wörgötter F (2015). The use of Hebbian cell assemblies for nonlinear computation. Sci Rep.

[180] Thangarasa V, Miconi T, Taylor GW (2019). Differentiable Hebbian plasticity for continual learning. International conference on machine learning (ICML) adaptive and multitask learning: Algorithms & Systems (AMTL) workshop.

[181] Thompson WR, Heron W (1954). The effects of restricting early experience on the problem-solving capacity of dogs. Can J Psychol.

[182] Tinbergen N (1942). An objective study of the innate behaviour of animals. Bibl. Biotheoret.

[183] Tolman EC (1927). Habit formation and higher mental processes in animals. Psychol Bull.

[184] Tomasello R, Garagnani M, Wennekers T, Pulvermüller FA (2018). Neurobiologically constrained cortex model of semantic grounding with spiking neurons and brain-like connectivity. Front Comput Neurosci.

[185] Tonegawa S, Liu X, Ramirez S, Redondo R (2015). Memory engram cells have come of age. Neuron.

[186] von Senden M. Raum- und Gestaltauffassung bei Operierten Blindgeborenen. Leipzig: Barth; 1932. [Reprinted in 1960 as Space and Sight: The perception of space and shape in the congenitally blind before and after operation. Translated by Peter Heath, with appendices by A. H. Riesen, G. J. Warnock and J. Z. Young. Glencoe, Illinois, The Free Press.

[187] Wallace DJ, Kerr JND (2010). Chasing the cell assembly. Curr Opin Neurobiol.

[188] Weiss P (1941). Autonomous versus reflexogenous activity of the central nervous system. Proc Am Philos Soc.

[189] Woodworth RS (1938). Experimental Psychology.

